# Arabic text preprocessing for dynamic searchable symmetric encryption: An empirical evaluation and preprocessor selection framework

**DOI:** 10.1371/journal.pone.0351281

**Published:** 2026-09-21

**Authors:** Kholoud Saad Al-Saleh

**Affiliations:** Department of Information Technology, College of Computer and Information Sciences, King Saud University, Riyadh, Saudi Arabia; University of Anbar, IRAQ

## Abstract

Dynamic Searchable Symmetric Encryption (DSSE) enables keyword search over encrypted data without revealing plaintext to the server. Arabic morphological richness — where a single root generates dozens of surface forms — creates substantial challenges for encrypted search: unprocessed vocabularies inflate encrypted-index size and transmission cost, and limit retrieval recall by failing to match morphological variants. This paper presents the first empirically grounded preprocessor selection framework for Arabic DSSE, derived from a systematic evaluation of four Arabic preprocessing strategies — the Khoja stemmer, ISRI stemmer, Lucene Arabic Analyzer, and Farasa segmenter — against a normalization-only baseline within the Incidence Matrix DSSE (IM-DSSE) scheme on the Khaleej Arabic news corpus. We evaluate vocabulary size, search latency, search quality, and retrieval breadth on an annotated 1,400-document corpus using 56 benchmark queries, and assess scalability on cloud infrastructure across corpus sizes up to 45,500 documents. Stemming reduces vocabulary by up to 83% relative to the normalization-only baseline, proportionally reducing encrypted-index size and transmission cost. We find that per-query search latency is driven primarily by result-set size rather than vocabulary size: the normalization-only baseline is fastest per query because it matches the fewest documents, while preprocessors that broaden retrieval incur higher latency. At scale, all five configurations — including the normalization-only baseline — operate successfully to 45,500 documents, and the result-set-size effect on latency persists. In terms of search quality, light stemming and morphological segmentation achieve the best trade-off between retrieval precision and recall, while root-based stemmers sacrifice precision without commensurate recall gains. Based on these findings, the framework provides actionable, evidence-based guidelines mapping deployment requirements — memory scalability, search latency, search quality, and retrieval breadth — to concrete preprocessor choices for Arabic DSSE system designers.

## 1. Introduction

Cloud computing has fundamentally altered how organizations store and retrieve sensitive documents. Outsourcing document collections to third-party cloud servers offers scalability and cost efficiency, but introduces a fundamental security risk: data encrypted at rest cannot be searched without first decrypting it, exposing plaintext to an untrusted server. Searchable Symmetric Encryption (SSE) [1] resolves this tension by enabling keyword search directly over encrypted data — the server identifies relevant documents given a cryptographic search token without ever learning the query keyword or document content.

SSE was later extended to Dynamic SSE (DSSE) to allow the addition and deletion of the documents in the document collection after the preliminary setup [2]. Then Hoang et al. [3] introduced the Incidence Matrix DSSE (IM-DSSE). IM-DSSE creates a binary encrypted incidence matrix over keyword–document pairs. The scheme’s memory usage increases in proportion to the number of unique keywords m and the number of documents n, making vocabulary size an essential variable for realistic deployment. IM-DSSE has been widely adopted as a practical DSSE framework, offering simultaneous forward privacy, backward privacy, and size-obliviousness with open-source availability, making it a suitable platform for systematic evaluation.

DSSE has matured into a recognized discipline with sophisticated models enabling secure search and retrieval over encrypted data saved on untrusted cloud servers [4,5]. While DSSE schemes assume a preprocessed keyword index as input — typically obtained through tokenization, stop-word removal, and keyword extraction — the choice of preprocessing pipeline is rarely examined in the literature and is treated as an implementation detail outside the cryptographic scope of SSE schemes [4, 5].

Arabic presents exceptional challenges for encrypted search that have received no systematic attention in the DSSE literature. Arabic is a morphologically rich, root-and-pattern language in which a single three-letter root can generate dozens of inflected surface forms through the attachment of prefixes, suffixes, and clitics. Searching for the token كتب (wrote) will not retrieve documents containing كتابة (writing) or كتبوا (they wrote) unless those inflectional variants are normalized to a common index token before encryption [6,7]. In conventional plaintext Arabic Information Retrieval (AIR), morphological preprocessing — including stemming, light stemming, and morphological segmentation — addresses this vocabulary mismatch by reducing surface forms to a canonical representation before indexing [6–8]. However, introducing morphological preprocessing into an encrypted search framework involves trade-offs that do not arise in plaintext IR: more aggressive normalization reduces the encrypted index vocabulary and improves search latency, but may introduce false positives that degrade retrieval precision. To the best of our knowledge, no prior work has examined this trade-off empirically within a deployed DSSE system [4,5]. This gap is technically significant: Arabic digital content is growing rapidly across government, legal, healthcare, and news domains; these documents increasingly require encrypted storage with search capability; and Arabic’s morphological complexity causes unprocessed encrypted indexes to grow to tens of thousands of unique tokens, imposing severe memory and latency costs on DSSE systems.

A major design choice when assessing preprocessing techniques for searchable encryption involves determining the rule of relevance. Within this paper, relevance is defined in relation to inflectional tokens. A document’s applicability to a query term is dependent upon the presence of the term itself or an inflectional variant of it. These variants include modifications in number, gender, definiteness, or case marking, representing the identical lexical item in varied grammatical forms. Notably, this definition remains neutral to preprocessing methodologies, remaining objective about the accuracy of specific stemmers in identifying inflectional demarcations and avoiding favoring derivational over-conflation. Under this rule, root-based stemmers such as Khoja [9] and ISRI [10] will retrieve documents containing derivationally related but lexically distinct forms — for example, شاعر (‘poet’) for a query on شعر (‘poetry’) — which represent false positives and decrease precision scores. This is a planned element of the evaluation design: it quantifies derivational over-conflation as a measurable precision cost, allowing direct comparison across preprocessors on a shared ground truth.

This paper addresses that gap by asking four research questions (RQs). RQ1: How does the choice of Arabic morphological preprocessor affect encrypted index vocabulary size and memory scalability in IM-DSSE? RQ2: How does preprocessor choice affect end-to-end search latency? RQ3: How does preprocessor choice affect search quality under a preprocessor-neutral relevance criterion? RQ4: How does preprocessor choice affect retrieval breadth — the number of documents returned per query, and the precision implications of broader retrieval? We evaluate four Arabic preprocessing strategies against a normalization-only baseline within a deployed IM-DSSE system on the Khaleej Arabic news corpus [11]. Search quality and per-query performance are measured on an annotated 1,400-document subset, using 56 benchmark queries across seven topical categories and a manually annotated relevance pool of 3,325 document–query pairs; scalability is then assessed on cloud infrastructure (AWS EC2) across corpus sizes ranging from 1,000–45,500 documents, the full corpus. Our contributions are:

1 The first empirically grounded preprocessor selection framework for Arabic DSSE, mapping application requirements — memory scalability (RQ1), search latency (RQ2), search quality (RQ3), and retrieval breadth (RQ4) — to concrete, evidence-based preprocessor choices (Section 5.4).

A systematic evaluation of four Arabic preprocessing strategies against a normalization-only baseline within IM-DSSE, measuring search latency, retrieval breadth, vocabulary size, and search quality on an annotated 1,400-document corpus (56 benchmark queries), and search latency and retrieval breadth at scale on cloud infrastructure — constituting the empirical evidence base for the selection framework.

A cloud-based scalability evaluation of all five configurations across corpus sizes up to 45,500 documents, establishing that per-query latency is driven primarily by result-set size rather than vocabulary size and that this effect persists at scale.

Qualitative analysis of three preprocessor-specific failure modes — loanword failure (Khoja on بيانات ‘data’, F1 = 0.000), over-reduction (ISRI on صلاه ‘prayer’, F1 = 0.047), and root conflation (ISRI on حاسوب ‘computer’, 277 retrieved vs. 4 for light stemmers) — with concrete implications for domain-specific preprocessor selection.

A benchmark keyword set of 56 queries across seven topical categories of the Khaleej corpus, provided in S1 Appendix for reproducibility.

The remainder of this paper is organized as follows. Section 2 provides background on searchable symmetric encryption and Arabic morphological preprocessing. Section 3 describes the experimental methodology, including the IM-DSSE system architecture, the Khaleej corpus, the benchmark query set, and the relevance annotation procedure. Section 4 presents the results across four dimensions: vocabulary size and memory scalability, search latency, retrieval breadth, and search quality. Section 5 discusses the findings and derives a preprocessor selection framework with evidence-based deployment guidelines. Section 6 concludes the paper and identifies directions for future work.

## 2. Background and related work

### 2.1. Searchable symmetric encryption

Searchable Symmetric Encryption was first introduced by Song et al. [1], who built a scheme enabling sequential search over encrypted data. Then Curtmola et al. [12] proposed the inverted index-based method and defined the formal security model for SSE, differentiating between adaptive and non-adaptive adversaries and introducing the concepts of search pattern and access pattern leakage. Chase and Kamara [13] expanded SSE to structured data. Then Kamara et al. [2] introduced DSSE to allow the addition and deletion of the documents in the document collection after the preliminary setup. Kamara and Papamanthou [14] introduced a Parallel DSSE scheme using a Keyword Red-Black (KRB) tree data structure. Later, Hoang et al. [3] introduced the IM-DSSE framework using incidence matrices and hash tables and provided an open source implementation written in C++ [15]. IM-DSSE stores an encrypted incidence matrix I of dimensions m × n, where m is the number of unique keywords and n is the number of documents. Each cell I[i,j] encodes, after XOR encryption with a pseudo-random value derived from a keyed hash function, whether keyword i appears in document j. Search proceeds in O(n) time per query: the client issues a row key for keyword i and the server decrypts row i to recover the matching column indices. The scheme supports constant-time update operations. Its principal limitation is the O(mn) storage cost, which makes vocabulary size a critical parameter and directly motivates vocabulary reduction through preprocessing. However, a critical limitation of IM-DSSE and related incidence matrix constructions is that they do not address the preprocessing layer: Hoang et al. assume a fixed vocabulary without examining how vocabulary size is determined or how different preprocessing strategies affect system performance. This leaves an important design decision unaddressed in the literature. In this paper we use the IM-DSSE scheme and single keyword queries. While more recent DSSE constructions have advanced the state of the art in security and functionality — including forward-private schemes such as Σoφoς [16], forward and backward-private schemes such as ROSE [17], and conjunctive query schemes — the preprocessing challenge studied in this paper is not specific to IM-DSSE. Any DSSE scheme that builds an inverted index or incidence matrix faces the same vocabulary inflation problem when applied to Arabic text: the number of unique keywords determines the index size, which affects both storage cost and search latency regardless of the specific cryptographic construction. The specific latency values reported in this study are particular to IM-DSSE’s O(n) search complexity, but the relative ordering of preprocessors — and the core trade-off between vocabulary reduction and retrieval precision — is a property of the preprocessing layer, not the cryptographic layer, and is therefore expected to transfer to other DSSE constructions. IM-DSSE was selected for this study because it is openly specified and well-characterized in the literature, and because its incidence matrix structure makes the relationship between vocabulary size and system performance explicit and measurable. The reference implementation released by Hoang [15] is written in C++. For this study the scheme was implemented in Java, so that the Arabic preprocessing libraries evaluated here — all of which are Java-based — could be integrated directly into the client without inter-language bridging. The implementation follows the scheme as specified by Hoang et al. [3]; the cryptographic construction is unchanged. Evaluating preprocessing within forward-private and conjunctive DSSE constructions represents an important direction for future work.

For a comprehensive treatment of SSE search functionalities, privacy leakages, and defenses, we refer the reader to the recent survey by Li et al. [5].

### 2.2. Arabic morphology and information retrieval

Arabic is a Semitic language with root-and-pattern morphology. Most Arabic words derive from a three- or four-letter root through the application of a morphological pattern, yielding a stem, to which prefixes and suffixes are attached. The root ك-ت-ب (‘write’) alone underlies كتب (wrote), كاتب (writer), مكتوب (written), مكتبة (library), كتابة (writing), وكتبوا (and they wrote) and many other forms. This richness means that a corpus of 1,400 Arabic news documents may contain over 76,000 unique word forms, compared to roughly 10,000–15,000 for an equivalent English corpus.

Arabic IR has been studied extensively within the Text Retrieval Conference (TREC) cross-language track [18]. Larkey and Connell [19] reported the first systematic comparison of Arabic stemmers at TREC-10, finding that light inflectional stemming outperformed root-based approaches for retrieval effectiveness. Larkey et al. [7] confirmed this finding using the TREC Arabic corpus of 383,872 documents and 25 queries, establishing that light stemmers — which normalize inflectional variants without aggressive derivational conflation — consistently outperform heavy root-based approaches in precision-recall balanced evaluation. Abu El-Khair [8] established the practice of treating diacritic removal and character normalization as a minimal preprocessing baseline equivalent to English lowercasing.

To ensure a fair comparison across preprocessors, relevance in this work is defined at the inflectional token level: a document is judged relevant to a query if it contains the query keyword or any inflectional surface variant of it, independently of which preprocessor produced the index. This preprocessor-neutral ground truth prevents any single preprocessing strategy from having an inherent advantage in the relevance assessment. Critically, while prior Arabic IR studies have established the superiority of light stemming over root-based approaches for retrieval effectiveness, none have examined these tradeoffs in an encrypted setting where vocabulary size directly determines memory consumption and index transmission cost — a fundamentally different optimization landscape from plaintext retrieval.

### 2.3. Arabic preprocessing tools

The four stemmed preprocessors and the normalization-only baseline evaluated in this paper are described below.

**Normalization-Only Baseline** (referred to throughout as NoPreprocessing) applies a sequence of five character-level normalization operations without any morphological analysis, making it the Arabic equivalent of English lowercasing in conventional SSE: (1) diacritic removal — all Arabic short vowels (tashkeel, Unicode range U + 064B to U + 065F) are stripped; (2) alef normalization — the four alef variants (أ, إ, آ, ٱ) are unified to the bare alef (ا); (3) yaa normalization — alef maqsura (ى) is mapped to yaa (ي); (4) taa marbuta normalization — taa marbuta (ة) is mapped to haa (ه); (5) tatweel removal — the elongation character kashida (ـــ, U+0640) is deleted. This treatment follows El-Khair [8] and Larkey et al. [7], who establish character normalization as the minimal preprocessing floor for Arabic IR.

**The Khoja Stemmer** [9] performs root extraction by removing prefixes and suffixes and then matching the residual against a lexicon of Arabic trilateral and quadrilateral roots. It was one of the earliest publicly available Arabic stemming tools and has been used as a standard research baseline across Arabic IR evaluations including the TREC Arabic track.

**The ISRI Stemmer** [10] applies a sequence of prefix and suffix stripping rules without requiring a root lexicon. The authors evaluated ISRI against Khoja on the TREC-2001 Arabic collection and found equivalent retrieval performance, establishing ISRI as an effective root-based stemmer that does not depend on dictionary coverage. ISRI also includes a built-in list of 60 Arabic stop words.

**The Lucene Arabic Analyzer** [20] implements the light stemming algorithm of Larkey et al. [6], combined with stop-word removal. Their stemmer, Light10, was subsequently incorporated into the Apache Lucene search library as its Arabic Analyzer, making it the most widely deployed Arabic light stemming implementation in production search systems.

**The Farasa Segmenter** [21] is a Support Vector Machine-based segmentation tool developed at the Qatar Computing Research Institute that decomposes Arabic words into prefix, stem, and suffix morphemes. Abdelali et al. [21] benchmarked Farasa against Khoja-based stemmers and MADAMIRA [22] on information retrieval tasks, finding that Farasa matches or exceeds the state of the art while being more than an order of magnitude faster. In this work, Farasa’s segmentation output is used directly, with stem morphemes retained as indexing tokens.

Arabic morphological preprocessing encompasses a broad range of techniques including character normalization, diacritic removal, stop-word removal, light stemming, root-based stemming, and morphological segmentation [7,23]. This study evaluates complete preprocessing pipelines as they would be deployed in practice — each preprocessor bundles its own combination of normalization, stop-word handling, and morphological reduction. Khoja, for example, includes explicit stop-word removal alongside root extraction [23], while Lucene integrates stop-word filtering within its light stemming analysis chain. This end-to-end pipeline evaluation reflects realistic DSSE deployment conditions more faithfully than isolated evaluation of individual preprocessing steps. The four stemmed preprocessors were selected to cover the three main paradigms of Arabic morphological preprocessing identified in the literature: root-based stemming (Khoja, ISRI), light stemming (Lucene), and morphological segmentation (Farasa) [6,7,21]. While some literature classifies segmentation-based approaches within the broader light stemming category [23], this paper follows Abdelali et al. [21] in treating morphological segmentation as a distinct paradigm, as it preserves morpheme boundaries rather than discarding affixes. Khoja and ISRI both perform root extraction but differ on dictionary dependency: Khoja requires lexicon lookup to validate a root, while ISRI proceeds through stripping rules alone. Taghva et al. [10] designed ISRI specifically as a dictionary-free alternative to Khoja and evaluated both under identical TREC-2001 conditions, making this pair a controlled comparison within the same root-extraction paradigm.

Lucene represents the light stemming paradigm. Larkey et al. [7] showed that light stemmers outperformed root extraction for retrieval on TREC-2001 Arabic data, and Larkey et al. [6] confirmed this finding on broader TREC data. Because the Lucene Arabic Analyzer is a direct Java implementation of Light10 and the most widely used Arabic search tool in production deployment, it provides the most practically relevant representative of the light stemming paradigm.

Farasa represents the most recent paradigm — morpheme segmentation — and was included because Abdelali et al. [21] explicitly evaluated it on IR tasks and reported state-of-the-art effectiveness combined with the lowest computational cost of any tool in their comparison.

Excluding more recent deep learning-based morphological analyzers was a deliberate design decision grounded in the specific constraints of DSSE deployment. Our selection is organized by preprocessing paradigm rather than by individual tool: the four preprocessors represent the three paradigms established in the Arabic IR literature — root-based stemming (Khoja, ISRI), light stemming (Lucene), and morphological segmentation (Farasa). We deliberately did not include full morphological analyzers and disambiguators — for example MADAMIRA [22] and CAMeL Tools [24] — or neural language-model taggers such as AraBERT [25] and other BERT-based analyzers (e.g., CAMeLBERT [26], MARBERT [27]), for reasons specific to DSSE deployment rather than to any single tool. First, these systems are designed for rich morphological disambiguation (producing lemmas, diacritics, part-of-speech, and full feature analyses) rather than the token-normalization that encrypted keyword search requires; their additional output does not translate into a different search token and therefore adds cost without retrieval benefit. Second, they impose deployment burdens that the selected tools do not: neural taggers are deep learning models whose inference is slow on CPU and practical only with GPU acceleration; Python-based toolkits (CAMeL Tools, AraBERT) expose no standalone Java library and require inter-language bridging with the Java-native IM-DSSE client; and heavyweight analyzers such as MADAMIRA, although Java-based, carry large memory footprints (on the order of gigabytes of heap) and restrictive licensing that are ill-suited to a lightweight client-side preprocessor replicated on every user device. In a DSSE system, preprocessing is performed client-side during both index construction and query time: every search query must be preprocessed before a search token is generated and sent to the server. This means the preprocessor executes on the client device, which in practice is consumer hardware without GPU acceleration. Neural models with inference times of hundreds of milliseconds per query would dominate end-to-end search latency, making them impractical for interactive search. This constraint cannot be avoided by preprocessing the corpus offline at index construction: searchable symmetric encryption requires that the same preprocessing be applied to queries and documents so that a query token matches the index (Section 3.1), so a tool that is too slow at query time is unusable regardless of its index-time cost. Together, the selected tools span the full performance spectrum reported for these paradigms in prior TREC Arabic IR evaluations [6,18], providing coverage of the deployable design space without requiring GPU infrastructure or heavyweight external analyzers. Deep learning-based approaches represent a distinct future direction that requires a separate evaluation framework beyond the scope of this work.

### 2.4. Research gap and positioning

The literature reviewed above reveals a clear and persistent gap. SSE and DSSE research has produced increasingly sophisticated cryptographic constructions, but has uniformly assumed English or language-agnostic preprocessing — to the best of our knowledge, no prior SSE or DSSE study has systematically evaluated the impact of Arabic morphological preprocessing on encrypted search performance. Arabic IR research, conversely, has extensively compared stemming approaches for retrieval effectiveness, but exclusively in plaintext settings where vocabulary size has no direct cost implication. The two bodies of literature have developed in isolation: IR researchers have no reason to consider encryption overhead, while cryptography researchers have no reason to consider morphological variation. The consequence is that a practitioner deploying Arabic DSSE today has no empirical basis for choosing among available preprocessing tools: the tradeoffs between vocabulary reduction, search latency, retrieval breadth, and search quality in an encrypted setting are entirely unknown. Furthermore, prior Arabic IR evaluations — while establishing that light stemming generally outperforms root-based approaches for retrieval effectiveness — have not examined the memory scalability implications of vocabulary size in resource-constrained deployment environments, nor have they evaluated preprocessing under the specific constraint that the preprocessor must run client-side on consumer hardware. The present study directly addresses this gap by providing the first empirical evaluation of Arabic preprocessing within a deployed DSSE system, covering all four dimensions simultaneously and deriving a framework that translates the findings into actionable deployment guidance.

Table 1 positions the present study against representative prior work to support the novelty claim. To the best of our knowledge, no prior study combines Arabic text with DSSE evaluation, preprocessor comparison, and a manually annotated relevance assessment.

**Table 1:** Comparison of the present study with representative prior work on SSE/DSSE and Arabic IR.

Study	DSSE Scheme	Arabic Text	Preprocessor Comparison	Relevance Annotation	Latency Measured	Selection Framework
Song et al. [1] 2000	Sequential SSE	No	No	No	No	No
Kamara et al. [2] 2012	DSSE (dynamic)	No	No	No	No	No
Hoang et al. [3] 2021	IM-DSSE	No	No	No	Yes	No
Larkey et al. [7] 2002	None (plaintext IR)	Yes	Yes	TREC judgments	No	No
Abdelali et al. [21] 2016	None (plaintext IR)	Yes	Yes (Farasa)	TREC judgments	No	No
**This study 2026**	**IM-DSSE**	**Yes**	**Yes (5 tools)**	**Yes (manual, κ = 0.956)**	**Yes**	**Yes**

Abbreviations: SSE, Searchable Symmetric Encryption; DSSE, Dynamic SSE; IR, Information Retrieval; IM-DSSE, Incidence Matrix DSSE; κ, Cohen’s kappa inter-annotator agreement.

## 3. Methodology

### 3.1. System architecture

We implement and evaluate all experiments within a Java implementation of the IM-DSSE scheme of Hoang et al. [3]. The system consists of a Java client that performs local encryption and keyword extraction, and a Java server that stores the encrypted index and responds to search tokens. Client and server communicate over a TCP socket using Java object serialization; to transmit the encrypted index without exhausting memory, the bit-packed matrix rows (Section 3.4) are streamed in chunks rather than as a single object. The system operates in two distinct phases. In the index construction phase, the client reads the plaintext document collection, applies the selected Arabic preprocessing pipeline to extract normalized keyword tokens, constructs a binary incidence matrix recording keyword–document co-occurrences, and encrypts each cell using AES before transmitting the encrypted index and encrypted documents to the server. In the search phase, the client preprocesses the query keyword using the same pipeline, generates a cryptographic trapdoor using a Pseudorandom Function (PRF), and transmits it to the server. The server performs index lookup using the trapdoor without decrypting any document content, and returns the matching encrypted documents and their identifiers to the client, which decrypts them locally using the secret key. The encrypted incidence matrix is stored as a dense bit-packed structure, with each cell occupying a single bit per plane (Section 3.4); because the XOR-encrypted matrix is not sparse, this representation is substantially more memory-efficient than a per-entry structure and is what enables scaling to larger corpora. Each cell of the incidence matrix is encrypted using a keyed PRF and a random oracle, following the construction of Hoang et al. [3]. Concretely, the client derives a per-row key rᵢ = PRF(k_3_, i ‖ c) using HMAC-SHA256, where c is the row’s counter, and masks each cell with a single bit obtained by applying a random oracle to (rᵢ, j, c); the stored value bit is the XOR of this mask with the plaintext co-occurrence bit, while the accompanying state bit records whether the cell was last written by an update or by a search, which determines which row key decrypts it. A search trapdoor consists of the row index i together with rᵢ, which enables the server to unmask that row alone and identify the matching columns; a second row key is included so that the row is re-encrypted under a fresh counter after each search. Document contents are encrypted independently under AES-CTR with a key that never leaves the client, so returned files remain opaque to the server.

**Fig 1:**
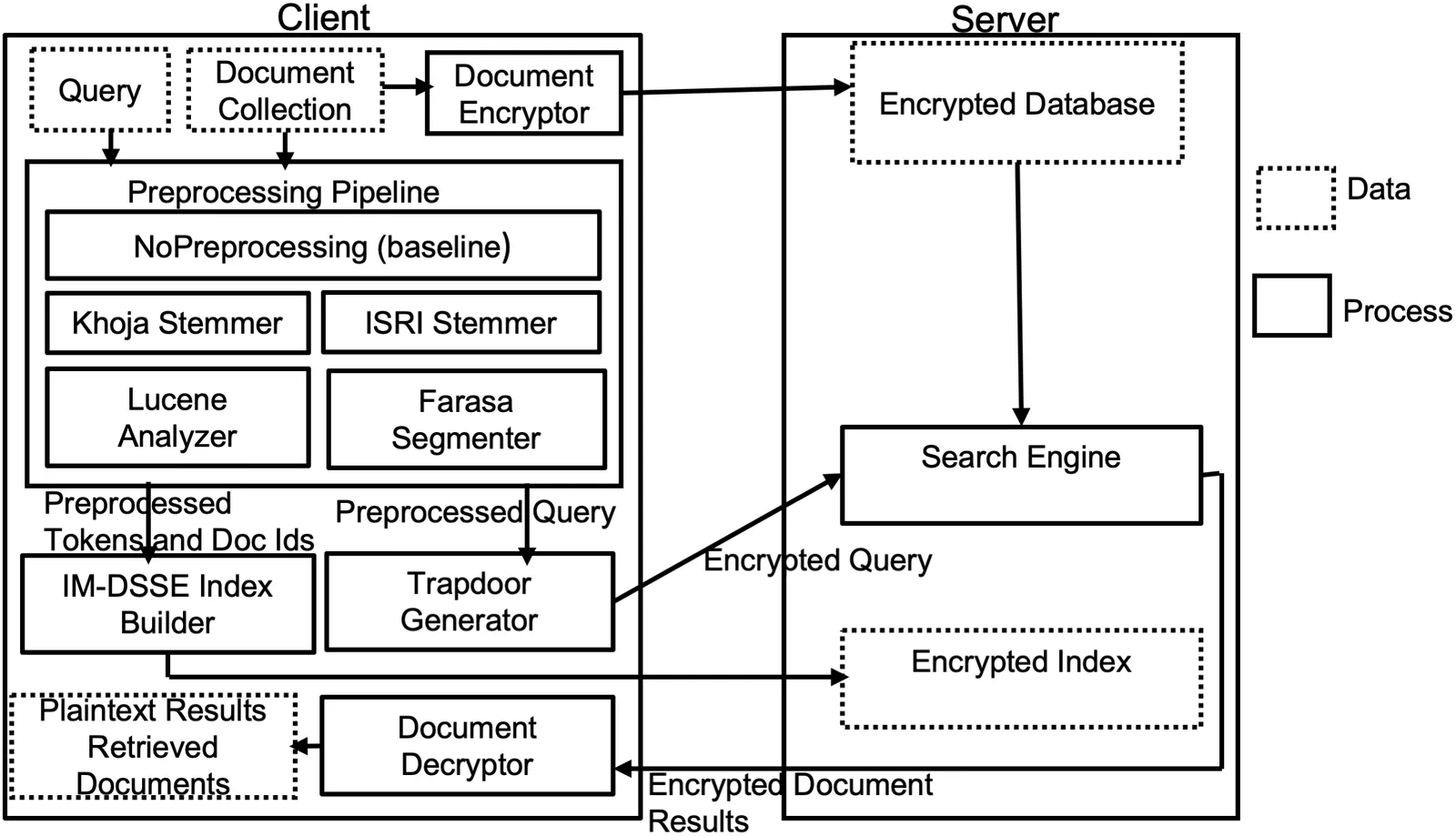
IM-DSSE system architecture for Arabic encrypted search evaluation. The client applies the selected Arabic preprocessing pipeline during both index construction and query processing; the server stores the encrypted incidence matrix and answers trapdoors over it, learning only which documents match a given query.

This construction ensures that the server learns nothing about the plaintext, keyword, or document content beyond the access pattern — that is, which encrypted documents match a given trapdoor — which is the standard leakage profile of IM-DSSE [3]. The Arabic preprocessor is modularly integrated into the client’s keyword extraction pipeline. Before indexing and before issuing each search query, text is processed through the selected preprocessor to extract the set of normalized keyword tokens. The server has no access to the preprocessor or to any plaintext: it receives only cryptographic tokens and returns encrypted file objects. Because the preprocessor operates identically on both the indexing side and the query side, the same preprocessing strategy must be applied consistently during both phases. This symmetry requirement is fundamental to SSE correctness: a trapdoor generated from a Farasa-segmented query token will only match index entries built from Farasa-segmented document tokens. The modular architecture therefore enables a controlled comparison in which only the preprocessing strategy varies across experimental conditions, while all cryptographic parameters remain fixed. Fig 1 illustrates the full system architecture.

**Fig 2:**
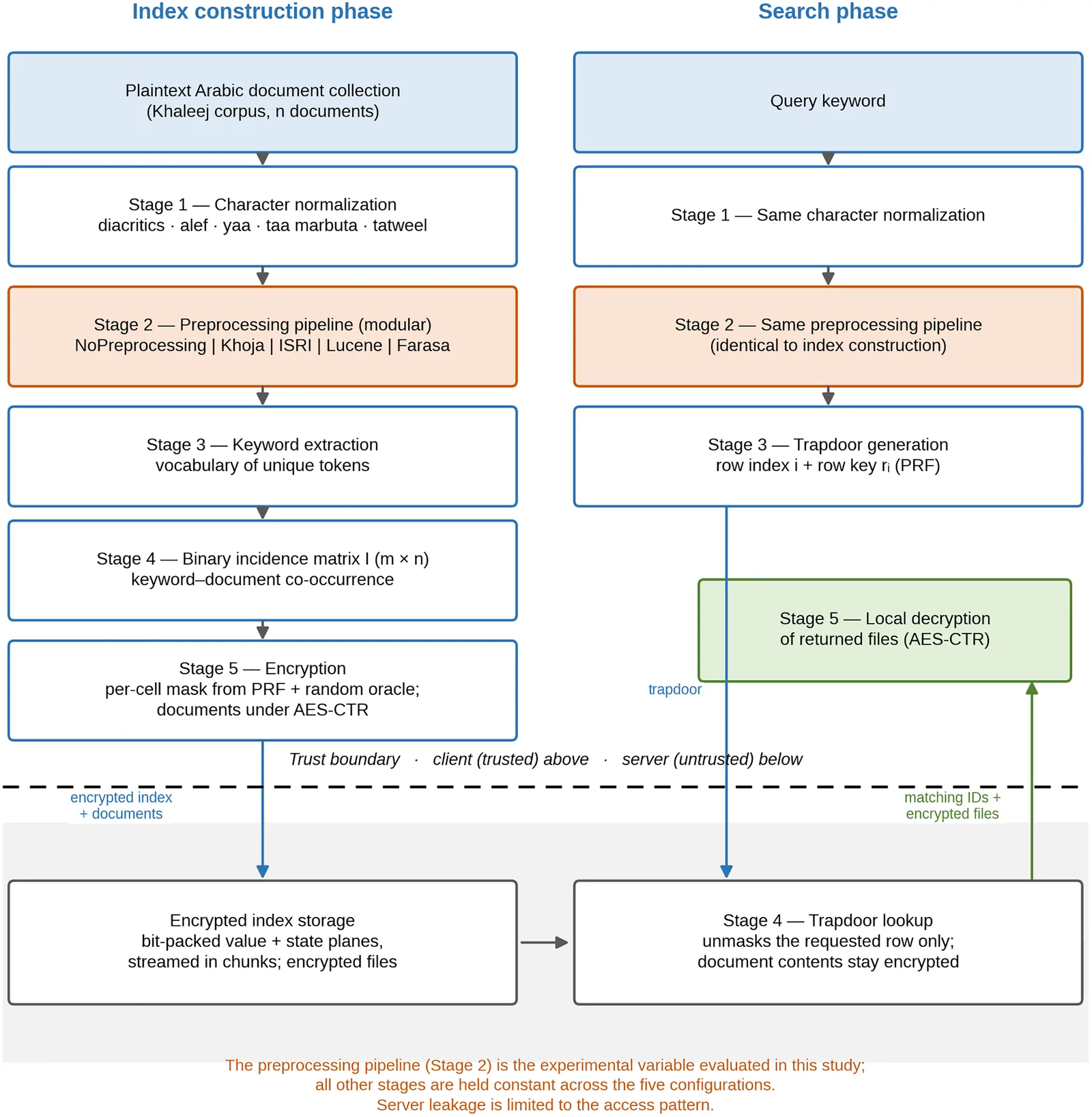
Design stages of the Arabic text preprocessing and DSSE pipeline. The index construction phase (left) and the search phase (right) apply identical character-normalization and preprocessing stages. The client performs all plaintext handling and key management; the untrusted server stores the bit-packed encrypted index and answers a trapdoor by unmasking only the requested row, learning which documents match but not the keyword or any document content. Stage 2 the preprocessing pipeline, is the experimental variable: five configurations — NoPreprocessing, Khoja, ISRI, Lucene, and Farasa — are compared with all other stages held constant.

Fig 2 decomposes this architecture into the ordered design stages executed in each phase, separating the operations performed on the trusted client from those performed by the untrusted server, and identifying the single stage — the preprocessing pipeline — that is varied in this evaluation.

### 3.2. Corpus

We use the Khaleej corpus [11], a collection of 45,500 Arabic news documents from Gulf Arabic news agencies, covering seven topical categories: Culture, Finance, Medical, Politics, Religion, Sports, and Technology. The corpus is encoded in UTF-8 and written in Modern Standard Arabic (MSA). For the search-quality evaluation, we sampled a balanced subset of 1,400 documents using stratified random sampling with seed 42 for reproducibility; the full 45,500-document corpus and intermediate subsets of it are used in the scalability evaluation (Section 3.4).

The choice of 1,400 documents is justified on three grounds. First, the sample is stratified across the seven Khaleej categories at exactly 200 documents per category, ensuring balanced topical representation. This stratification constraint fixes the sample size to a multiple of seven, and 1,400 (7 × 200) is the largest such balanced sample evaluated in this protocol. Second, 1,400 documents represent the maximum corpus size for a controlled, fair comparison: the 1,400-document corpus is the subset for which we constructed a manually annotated relevance pool (Section 3.6), enabling all five configurations to be evaluated on equal terms with human-verified ground truth; manual annotation at this scale (3,325 document–query pairs) was tractable, which it would not be at substantially larger corpus sizes. Third, for a study focused on comparative preprocessing behavior within a DSSE framework, 1,400 documents provide sufficient statistical power for the 56-query, 5-run benchmark (280 measurements per configuration, 1,400 total).

### 3.3. Benchmark query set

**Curated benchmark query set.** We constructed a benchmark of 56 Arabic queries distributed evenly across the seven Khaleej categories (8 queries per category), following the TREC-2002 design guideline of at least 50 evaluation queries [18].

**Scalability query pool.** For the scalability evaluation (Section 4.8), which measures efficiency rather than retrieval quality and therefore does not require manual relevance annotation, we constructed a larger pool of 400 Arabic queries. The pool comprises the 56 curated benchmark keywords described above, plus 344 high-frequency content words extracted automatically from the full Khaleej corpus: candidate words were ranked by document frequency and included if they appeared in at least 5 documents, contained at least 3 Arabic characters, and were not common Arabic function words (excluded via a stop-word list). This frequency-weighted composition approximates a realistic query workload while guaranteeing that the curated benchmark keywords are represented. The complete pool is provided as S1 File.

### 3.4. Experimental protocol

This study comprises two experimental protocols: a search-quality evaluation on the annotated 1,400-document corpus, and a scalability evaluation across corpus sizes up to 45,500 documents on cloud infrastructure. Both are described below.

**Search-quality evaluation protocol**. Each experimental session began with two warm-up queries drawn from the benchmark set, which were excluded from analysis to allow the Java Virtual Machine (JVM) to reach steady-state just-in-time (JIT) compilation. Each of the five preprocessors was then evaluated on the full 56-query benchmark with five timed runs per query, for 280 searches per preprocessor and 1,400 total measurements. For each search, we recorded: search token generation time (ms), network round-trip time (ms), total end-to-end latency (ms), result decryption time (ms), and number of documents returned. Per-query measurements were averaged across the five runs before statistical analysis.

The search-quality evaluation was conducted on an Apple MacBook Pro with Apple M1 system-on-chip (SoC) and 8 GB unified memory, running macOS 15.7.3 (Sequoia), using OpenJDK 11.0.29 with a JVM heap limit of 8 GB; the client and server ran as separate processes on this machine, communicating over the loopback interface. The encrypted index was transmitted from client to server using chunked serialization of the bit-packed matrix rows (see Index representation below).

**Software configuration.** The Khoja stemmer is integrated via the original Stem.java implementation [9] with the StemmerFiles lookup tables encoded in UTF-16 LE. The ISRI stemmer is integrated via the ISRI.java implementation [10] with its built-in 60-word-stop list. The Lucene Arabic Analyzer is integrated via Apache Lucene 9.4.2 (lucene-analyzers-common-9.4.2.jar) [20], using the ArabicAnalyzer class with its default Arabic stop-word list. The Farasa segmenter is integrated via the official Farasa Java API (farasa-2.1.jar) [21], using the Farasa.segmentLine() method with default configuration. All stop-word removal and stemming settings use each library’s default configuration to ensure reproducibility; no custom stop-word lists or stemming aggressiveness parameters were modified beyond the defaults.

**Index representation.** The encrypted incidence matrix is stored as dense bit-packed rows, with one bit per cell for the value plane and one for the state plane, matching the representation assumed in the original IM-DSSE design. An earlier version of our implementation stored the matrix as a per-entry hash map; because IM-DSSE encrypts each cell by XOR with a pseudorandom keystream, approximately half of all ciphertext cells are non-zero regardless of plaintext sparsity, so a per-entry representation incurs object-storage overhead of roughly two orders of magnitude relative to the information content of the matrix. All latency, retrieval-breadth, and scalability results reported in this paper were obtained with the corrected bit-packed implementation; the ranked result lists underlying the search-quality evaluation (Section 3.6) were exported from the earlier per-entry implementation. The correction affects memory consumption and absolute latency values. Result counts are identical across implementations and repeated benchmark runs for all 56 benchmark queries under NoPreprocessing, Khoja, Lucene, and Farasa; under ISRI, four queries (روايه, صحه, زكاه, تقنيه) exhibit small run-to-run variations in result count (within approximately 8% of the roughly 700 documents returned), independent of the index implementation. A re-export of the ranked result lists using the corrected implementation confirmed that the top-20 lists defining the annotation pool are identical for 276 of the 280 preprocessor–query combinations, the four ISRI queries noted above being the only exceptions; recomputing all quality metrics on the corrected rankings against the same relevance judgments changes no Table 2 value by more than 0.003 and alters no qualitative finding.

**Table 2:** Search quality metrics: mean Precision, Recall, and F1-score per preprocessor, averaged over 56 queries (1,400 documents, Khaleej corpus). Bold row indicates the normalization-only baseline.

Preprocessor	Precision	Recall	F1-Score
**NoPreprocessing**	0.993	0.384	0.537
Khoja	0.527	0.279	0.341
ISRI	0.556	0.277	0.349
Lucene	0.930	0.490	0.615
Farasa	0.922	0.468	0.599

Each metric is computed per query and averaged over the 56 benchmark queries (macro-averaging). The reported F1-score is the mean of the per-query F1-scores; it is therefore not equal to the harmonic mean of the mean Precision and mean Recall shown in this table.

**Implementation comparison.** For transparency, we summarize the two index implementations used over the course of this study and confirm that they differ only in physical data representation, not in cryptographic construction or retrieval semantics. Both realize the identical IM-DSSE scheme of Hoang et al. [3]: an m × n binary incidence matrix (m keywords × n documents) in which each cell is independently encrypted with a keyed pseudorandom function and a random oracle, and each search applies a PRF-derived trapdoor to the encrypted rows. The set of encryption operations, the trapdoor-generation procedure, and the index-lookup semantics are identical between the two implementations; only the in-memory layout of the encrypted matrix differs. The original implementation stored the matrix as a per-entry hash map, materializing one object per set cell. This representation is efficient only for sparse matrices, and the encrypted matrix is not sparse: after XOR encryption with the pseudorandom keystream, approximately half of all cells hold a one irrespective of plaintext sparsity. The map therefore retains on the order of m·n/2 entries, each costing approximately 100 bytes once the boxed key, cell object, and map node are counted, so resident memory grows as O(m·n) with a large constant; on 8 GB RAM this produced an out-of-memory condition for the baseline vocabulary at approximately 1,500 documents (Section 4.1). The corrected implementation stores each row as two contiguous bit-packed planes — one for the encrypted value bit I[i,j].v and one for the state bit I[i,j].st — each holding a single bit per cell in a long array of ⌈n/64⌉ words. The encrypted index therefore occupies 2·m·n bits plus per-row array overhead, and rows are allocated lazily so that memory scales with the number of keywords actually indexed rather than the configured maximum. At the full evaluation scale this amounts to approximately 5.5 GB for the normalization-only baseline (479,703 keywords across 45,500 documents; Section 4.8.1). By contrast, the per-entry model predicts approximately 5.9 GB at only 1,500 documents, consistent with the out-of-memory condition observed there; the bit-packed representation is therefore what permits indexing the complete corpus. Because the two implementations apply the same encryption to the same matrix and expose the same lookup interface, they return the same document sets for the same query, corpus, and preprocessor; the only observed exceptions are the four ISRI queries discussed above, whose variation arises in the preprocessing stage and is independent of the index representation. The correction therefore affects memory consumption and absolute latency only, leaving all retrieval and search-quality findings unchanged, as the robustness re-export above quantifies. Table 3 compares the two implementations: they differ only in physical memory layout, while the cryptographic construction, trapdoor generation, and lookup semantics are identical.

**Table 3:** Comparison of the original and corrected index implementations. Under ISRI, four queries (روايه, صحه, زكاه, تقنيه) show small run-to-run variation in result count, within approximately 8% of the roughly 700 documents returned.

Property	Original (per-entry)	Corrected (bit-packed)
Cryptographic construction	IM-DSSE [3]: keyed PRF + random oracle per cell	IM-DSSE [3]: keyed PRF + random oracle per cell (identical)
Trapdoor/ lookup semantics	PRF trapdoor; encrypted-row lookup	PRF trapdoor; encrypted-row lookup (identical)
Physical representation	One hash-map entry per set cell (ciphertext is ~ 50% ones, so not sparse)	Two bit-packed planes per row (value bit + state bit), ⌈n/64⌉ longs each
Memory model (encrypted index)	≈ m·n/2 entries × ~100 B; O(m·n) with large constant	2·m·n bits + per-row padding and array overhead; ≈ 5.5 GB for the baseline at m = 479,703, n = 45,500 (Section 4.8.1); rows allocated lazily
Largest corpus indexed	≈1,500 documents (baseline vocabulary, 8 GB RAM)	45,500 documents (full corpus, 24 GB server heap)
Returned result sets	Identical across the two implementations for all 56 queries under NoPreprocessing, Khoja, Lucene, and Farasa; four ISRI queries vary between runs independently of the implementation.	—

**Scalability experiment protocol.** The scalability evaluation (Section 4.8) was conducted on cloud infrastructure: an Amazon Web Services (AWS) EC2 r6i.2xlarge instance (8 vCPUs, 64 GB RAM, eu-central-1 region). The DSSE client and server were deployed as separate Java processes communicating over the TCP protocol described in Section 3.1, exercising the full client–server search pipeline. The server process was allocated a 24 GB JVM heap and the client 16 GB, corresponding to typical cloud-server and enterprise-workstation memory configurations; communication used the loopback interface (network round-trip time effectively zero), isolating computational scaling from network-transport effects. Five corpus sizes were evaluated: subsets of 1,000, 5,000, 10,000, and 25,000 documents sampled uniformly at random from the full Khaleej corpus with a fixed seed, plus the full corpus of 45,500 documents. For each of the 25 configuration–size cells, 20 warm-up searches were executed and discarded, followed by 280 timed search operations whose queries were drawn uniformly at random (with replacement) from the 400-query pool using a fixed random seed, ensuring an identical query sequence for a given preprocessor across all corpus sizes and full reproducibility.

### 3.5. Statistical analysis

We used the Wilcoxon signed-rank test [28] to assess the statistical significance of search latency differences between preprocessors, following the recommendation of Hull [29] for IR evaluation. The Wilcoxon test is non-parametric and appropriate for paired measurements that may not follow a normal distribution. Tests were conducted on per-keyword mean latencies: the five timed runs for each keyword were averaged, yielding 56 paired observations per comparison. We report W-statistics and two-tailed p-values, with significance thresholds at p < 0.05 (*), p < 0.01 (**), and p < 0.001 (***). All statistical analyses were performed with SciPy 1.17.1 (scipy.stats.wilcoxon) under Python 3.12. The same test was applied to search quality: pairwise comparisons of per-query F1-scores across all five configurations (ten comparisons, 56 paired observations each) are reported in Section 4.7 and S2 Appendix. For latency, ten pairwise comparisons are reported in total (four against the baseline, Table 4; six among the stemmed preprocessors, S2 Appendix); we note that eight of the ten remain significant under a conservative Bonferroni correction (α = 0.05/10 = 0.005), while the Khoja–Farasa and ISRI–Lucene comparisons are significant only at the uncorrected p < 0.05 level and should be interpreted with corresponding caution. Search-quality metrics (Section 3.6) are reported descriptively. The Wilcoxon test was selected over parametric alternatives (e.g., paired t-test) because search latency distributions in our benchmark are right-skewed due to occasional JVM garbage collection events, violating the normality assumption. It was preferred over the randomization test for its established use and interpretability in IR evaluation [29].

**Table 4:** Wilcoxon signed-rank test results: search latency vs. Normalization-Only baseline.

Comparison	W-statistic	p-value	Significance	Baseline advantage (ms)
NoPreprocessing vs. Khoja	258.5	< 0.001	***	+1.73
NoPreprocessing vs. ISRI	34.0	< 0.001	***	+5.87
NoPreprocessing vs. Lucene	16.0	< 0.001	***	+7.47
NoPreprocessing vs. Farasa	305.0	< 0.001	***	+0.91

Significance: * p < 0.05; ** p < 0.01; *** p < 0.001 (two-tailed, uncorrected). Tests were conducted on per-keyword mean latencies (n = 56 paired observations per comparison). Baseline advantage is the mean latency difference (ms) relative to the normalization-only baseline.

### 3.6. Search quality evaluation and relevance rule

**Metrics.** Search quality is assessed using three standard IR metrics: Precision (the proportion of retrieved documents that are relevant), Recall (the proportion of all relevant documents that are retrieved), and F1-score (the harmonic mean of Precision and Recall). All three metrics are computed per query and then averaged across the 56 benchmark queries; the reported F1-score is therefore the mean of the per-query F1-scores, and is not equal to the harmonic mean of the reported mean Precision and mean Recall. Rank-aware and classification-based measures are not reported, for two reasons intrinsic to the evaluation setting. First, IM-DSSE performs binary unranked retrieval: a search returns the complete set of matching documents simultaneously, with no relevance scores and no rank ordering. Rank-aware measures — Mean Average Precision (MAP), nDCG, and area under the ROC curve (AUC) — are therefore not meaningful, since any such value computed over the returned set would reflect only the arbitrary order in which documents are enumerated rather than retrieval effectiveness. Second, measures that require a true-negative count — accuracy and specificity — are uninformative in this setting. Documents outside the judged pool are treated as not relevant by convention (Section 4.7) rather than individually assessed, so the negative class is fixed by corpus size rather than established by evidence, and it dominates both measures: computed on this basis, accuracy ranges from 0.971 to 0.982 and specificity from 0.993 to 1.000 across the five configurations, compressing into the third decimal place differences that F1 resolves across a range of 0.341 to 0.615 [30]. Set-based Precision, Recall, and F1, computed per query and macro-averaged, are the standard and appropriate measures for this evaluation.

**Relevance criterion.** Relevance is defined at the token level, grounded in the token-matching semantics of SSE: a document is judged *relevant* to a query keyword if it contains the query keyword token itself or an inflectional surface variant of that token — meaning the same lexical item realized in a different grammatical form, such as a different number (singular/plural), gender (masculine/feminine), definiteness (definite/indefinite), or possessive suffix. Because the SSE server performs token matching rather than semantic retrieval, relevance is token-presence based and is independent of the word sense instantiated in the document: for example, a document containing شعر (‘poetry/hair’) in the sense of hair is *relevant* to a query on شعر (‘poetry’) in the sense of poetry, because the same token appears and would be correctly retrieved by the SSE system. Conversely, this definition excludes derivationally distinct lexical items, even if a particular stemmer conflates them with the query keyword. For example, a document containing only شاعر (poet) is *not relevant* for a query on شعر (poetry/hair). Although root-based stemmers such as Khoja may map both words to the same root token (ش-ع-ر), شاعر (‘poet’) is a derivationally distinct lexical item — an agent noun — not an inflectional variant of شعر (‘poetry’). A user searching for شعر (‘poetry’) who retrieves documents containing only شاعر (‘poet’) has not had their information need met, even if the system performed internally consistent root-based matching. This criterion therefore measures the cost of over-conflation as a precision penalty, which is the primary contribution of the search quality evaluation in Section 4.7.

This token-level relevance rule is preprocessor-neutral by design: it does not favor any specific preprocessor and does not assume that root-based conflation is correct. Under this rule, root-based stemmers such as Khoja and ISRI will systematically retrieve documents containing derivationally related but lexically distinct forms — for example, شاعر (‘poet’) for a query on شعر (‘poetry’) — which constitute false positives and reduce precision scores. This is an intended feature of the evaluation design: it quantifies the cost of derivational over-conflation as a measurable precision penalty, consistent with the evaluation methodology of the TREC Arabic track [18,19], in which human assessors judged documents against the original topic need rather than against any specific stemmer’s output. A preprocessor that maps many distinct words to the same root token — retrieving more documents — is not rewarded for breadth alone; it must retrieve documents that genuinely contain the original query keyword or a legitimate inflectional form of it.

**Annotation pool construction.** The annotation pool of 3,325 unique document–query pairs was constructed by depth-20 pooling: for each of the 56 queries, the top 20 documents returned by each of the five preprocessors were merged and duplicates removed, following standard TREC pooling practice. The pool covers all 56 queries, with a mean of 59.4 unique documents per query (range: 22–83). Because judgments are pool-based, Recall is measured against the pooled relevant set rather than against the full corpus. Human relevance judgments were collected by a primary annotator using a purpose-built browser-based annotation interface. Relevance was assessed using the token-level criterion defined above. To assess annotation reliability, a second annotator independently judged a stratified random subset of 665 document–query pairs (20.0% of the pool, 95 pairs per category), yielding Cohen’s κ = 0.956, confirming that the criterion is operationally unambiguous [31].

### 3.7. Ethics statement

This research is entirely computational, consisting of automated benchmarking of text preprocessing algorithms on a publicly available Arabic news corpus (Khaleej corpus). The relevance annotation task involved a single second annotator who assessed document relevance for inter-annotator agreement purposes; no personal data were collected, no sensitive information was processed, and participation was voluntary with full awareness of the study purpose. No ethical approval or informed consent was required for this type of computational research.

### 3.8. Use of AI tools

The author used Claude (Anthropic) as a writing- and analysis-support tool during the preparation and revision of this manuscript: for language editing; for assistance computing summary statistics and generating tables and plots from the author’s own experimental output files; and for assistance with code development and debugging. AnswerThis (answerThis.io) was used to support literature searching. All experimental data were produced by executing the author’s benchmark software on the corpus data; no AI tool was used to generate, fabricate, or alter any experimental measurement or result, or the data depicted in any figure. The author designed the study, conducted all experiments, made all analytical and interpretive decisions, and independently verified every reported statistic, table, and figure against the underlying data. The author takes full responsibility for the content of the article.

## 4. Results

### 4.1. Vocabulary size and memory scalability

**Fig 3:**
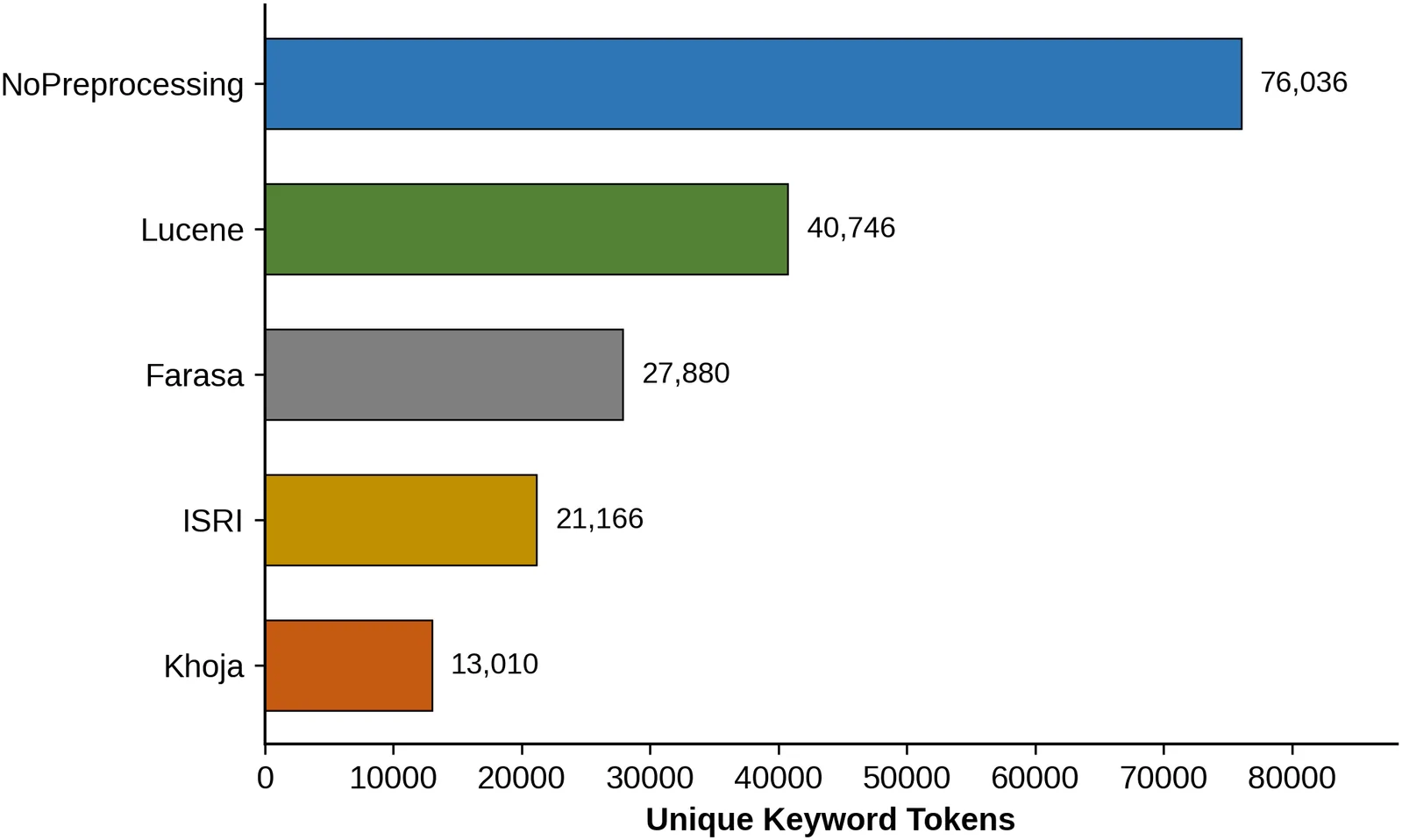
Encrypted index vocabulary size by preprocessing pipeline. Root-based stemmers achieve the greatest reduction; Khoja reduces vocabulary size by 82.9% relative to the normalization-only baseline (76,036 tokens). Vocabulary sizes (unique keyword tokens) are shown to the right of each bar. Khaleej corpus, 1,400 documents.

Fig 3 shows the vocabulary size produced by each pipeline. The difference is striking: Khoja reduces the index to 13,010 unique tokens, an 82.9% reduction relative to the normalization-only baseline of 76,036 tokens. Table 5 reports the number of unique keywords extracted by each preprocessor from the 1,400-document evaluation corpus, reflecting the encrypted index vocabulary size for each configuration. The normalization-only baseline produces 76,036 unique keyword tokens, the largest vocabulary, consistent with the expectation that unprocessed Arabic morphology yields the richest surface form inventory. Stemmed preprocessors reduce vocabulary size substantially: Lucene produces 40,746 tokens (46% reduction), Farasa 27,880 (63%), ISRI 21,166 (72%), and Khoja 13,010 (83%). These vocabulary counts directly determine the row dimension m of the IM-DSSE encrypted incidence matrix, making them the primary driver of memory consumption and index construction time.

**Table 5:** Unique keyword counts per preprocessor (1,400 documents, Khaleej corpus, stratified 200 docs/category).

Preprocessor	Unique Keywords	Reduction vs. Baseline
**NoPreprocessing**	76,036	—
Lucene	40,746	46%
Farasa	27,880	63%
ISRI	21,166	72%
Khoja	13,010	83%

These vocabulary differences have direct implications for encrypted index size. The row dimension m of the IM-DSSE incidence matrix equals the vocabulary size, so the normalization-only baseline (76,036 keywords) produces the largest index and Khoja (13,010 keywords) the smallest — an 83% reduction in index rows. In a bit-packed representation (Section 3.4), index memory is proportional to m × n bits, giving the baseline a correspondingly larger memory footprint and transmission cost than the stemmed configurations at any given corpus size. We note that an earlier per-entry implementation of the index exhibited an out-of-memory condition for the baseline at approximately 1,500 documents on 8 GB RAM; on investigation this proved to be an artifact of the per-entry storage overhead rather than an intrinsic limit of the scheme, since the XOR-encrypted matrix is not sparse. With the corrected bit-packed representation, all five configurations — including the normalization-only baseline — scale to 45,500 documents (Section 4.8). Khoja, with the smallest vocabulary, produces the most compact encrypted index and therefore the lowest memory and transmission cost at scale. The relationship between vocabulary size and encrypted index density is confirmed by non-zero entry counts: the baseline produces 53,220,955 matrix entries while Farasa produces 19,514,837, a 63% reduction consistent with its vocabulary reduction ratio.

**Fig 4:**
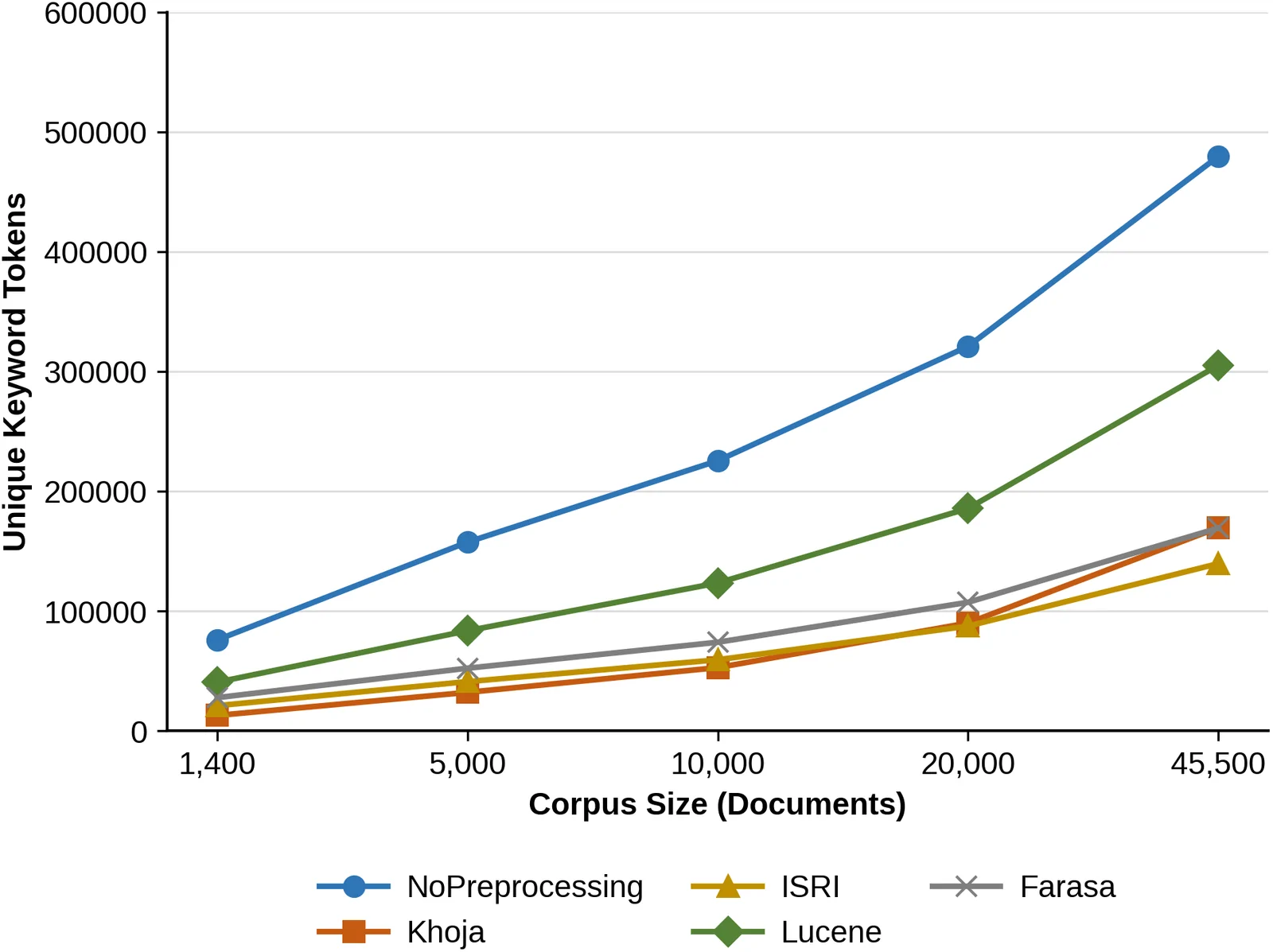
Vocabulary growth across five corpus sizes by preprocessing pipeline. All preprocessors exhibit sub-linear vocabulary growth; the normalization-only baseline grows 6.3× (from 76,036 to 479,703 tokens), the largest index of any configuration. Growth ratios for the stemmed preprocessors: Farasa 6.1 × , ISRI 6.6 × , Lucene 7.5 × , Khoja 13.1 × . Khaleej corpus, 1,400 to 45,500 documents.

Fig 4 shows how the vocabulary count extends across five corpus sizes, from 1,400–45,500 documents, illustrating how each preprocessor scales as the corpus grows. The normalization-only baseline grows from 76,036 tokens at 1,400 documents to 479,703 at 45,500 — a 6.3 × increase. Khoja shows the steepest relative growth (13.1×), reflecting the accumulation of rare roots from larger corpora; Farasa (6.1×), ISRI (6.6×), and Lucene (7.5×) grow at rates close to the baseline’s 6.3 × . In absolute terms, all four stemmed preprocessors maintain substantially smaller vocabularies than the baseline at every corpus size.

### 4.2. Search performance

Table 6 reports descriptive statistics for end-to-end search latency across all 56 queries and five timed runs per query (280 measurements per preprocessor), following two discarded warm-up queries per session. The normalization-only baseline achieves the lowest mean latency at 4.44 ms (SD = 5.54), followed by Farasa at 5.35 ms (SD = 5.46), Khoja at 6.16 ms (SD = 7.94), ISRI at 10.31 ms (SD = 10.20), and Lucene at 11.91 ms (SD = 7.29). This ordering reflects a central finding of this study: per-query search latency is driven primarily by the number of documents matched per query rather than by vocabulary size. The normalization-only baseline, which performs exact-form matching without morphological expansion, returns the fewest documents per query (47.2 on average; Table 7) and therefore incurs the least server-side row processing and client-side decryption. Root-based stemmers, which conflate many surface forms to a shared root, return substantially larger result sets (Khoja 235.5, ISRI 223.5 documents per query) and consequently exhibit higher decryption cost. Segmentation (Farasa) and light stemming (Lucene) occupy intermediate positions.

**Table 6:** Search latency statistics (ms) across 56 queries × 5 runs, 1,400 documents (with JVM warm-up).

Preprocessor	Mean	Median	SD	Min	Max
**NoPreprocessing**	4.44	3.00	5.54	1.00	73.00
Khoja	6.16	5.00	7.94	0.00	121.00
ISRI	10.31	7.00	10.20	1.00	106.00
Lucene	11.91	11.00	7.29	5.00	82.00
Farasa	5.35	4.00	5.46	1.00	78.00

**Table 7:** Mean documents returned per query (retrieval breadth), 1,400 documents.

Preprocessor	Mean	Median	SD	Max	vs. Baseline
**NoPreprocessing**	47.2	30.0	55.7	278	1.00×
Khoja	235.5	220.5	179.3	756	4.99×
ISRI	223.5	163.5	205.8	724	4.73×
Lucene	116.4	90.5	101.7	498	2.46×
Farasa	99.3	69.5	92.7	442	2.10×

Each keyword was executed five times following two discarded warm-up queries. The normalization-only baseline exhibits the lowest mean latency (4.44 ms) because its exact-form matching returns the smallest result sets (47.2 documents per query), minimizing both the number of encrypted index rows scanned and the volume of document identifiers decrypted. Root-based stemmers show higher latency: Khoja (6.16 ms) and ISRI (10.31 ms) conflate morphological variants to shared roots, matching far more documents per query (235.5 and 223.5 respectively) and thus incurring greater decryption cost. Farasa achieves the second-lowest latency (5.35 ms) by combining a moderate result-set size (99.3 documents per query) with efficient segmentation. Lucene shows the highest mean latency (11.91 ms) but the lowest variability relative to its mean (SD = 7.29 ms; coefficient of variation 0.61), indicating consistent and predictable performance relative to its typical latency across diverse query types.

The Khoja minimum of 0 ms reflects both a timer resolution effect and the keyword بيانات (data) returning no results after Khoja root extraction: with zero documents to decrypt, the operation completed in under 1 ms, below the resolution of the millisecond timer.

### 4.3. Statistical significance

Table 4 reports Wilcoxon [28] signed-rank test results comparing the normalization-only baseline against each stemmed preprocessor. The baseline achieves a small but statistically significant latency advantage over all four stemmed preprocessors: versus Khoja (W = 258.5, p < 0.001), ISRI (W = 34.0, p < 0.001), Lucene (W = 16.0, p < 0.001), and Farasa (W = 305.0, p < 0.001). The baseline advantage ranges from 0.91 ms (versus Farasa) to 7.47 ms (versus Lucene). These results confirm that, at the per-query level, morphological preprocessing does not reduce search latency; preprocessors that broaden retrieval by conflating morphological variants incur higher latency through larger result sets. This finding must be read alongside the search-quality results (Section 4.7): the baseline is fastest precisely because it retrieves the fewest documents, which also limits its recall.

Pairwise comparisons among the four stemmed preprocessors (reported in S2 Appendix) reveal that all pairwise differences are statistically significant at p < 0.05, with most pairs at p < 0.001, confirming a clear performance ordering for search latency in ascending mean: NoPreprocessing (4.44 ms), Farasa (5.35 ms), Khoja (6.16 ms), ISRI (10.31 ms), and Lucene (11.91 ms).

### 4.4. Retrieval breadth

Table 7 reports the mean number of documents returned per query, which serves as a system-level measure of retrieval breadth. Khoja returns the most documents on average (235.5, SD = 179.3), followed by ISRI (223.5, SD = 205.8), Lucene (116.4, SD = 101.7), Farasa (99.3, SD = 92.7), and NoPreprocessing (47.2, SD = 55.7). All stemmed preprocessors retrieve substantially more documents than the baseline, ranging from 2.10× (Farasa) to 4.99× (Khoja). The relationship between retrieval breadth and preprocessor aggressiveness — root-based stemmers retrieve more broadly than light stemmers or segmenters — is consistent with the expected behavior of each approach and with findings from the Arabic IR literature [7,19]. Whether this additional breadth reflects genuine recall improvement or over-conflation false positives is addressed in the search quality analysis (Section 4.7).

### 4.5. Category-level analysis

Table 8 breaks down mean search latency by topical category. Two findings emerge from the category-level data. First, per-category latency broadly tracks result-set size rather than preprocessing paradigm: categories whose queries match many documents (e.g., Culture, Finance) show higher latency across all preprocessors, while sparse categories (e.g., Technology) are fast for every preprocessor. The normalization-only baseline shows a moderate range of mean latency across the seven categories (2.2–10.5 ms, SD = 3.0 ms), comparable to Farasa (2.8–10.0 ms, SD = 2.6 ms) and Khoja (3.5–11.3 ms, SD = 2.7 ms), whereas ISRI (4.7–21.4 ms, SD = 6.7 ms) and Lucene (6.4–16.8 ms, SD = 3.5 ms) show wider variation driven by larger result sets in high-frequency categories. This pattern indicates that per-category latency primarily reflects retrieval breadth rather than the specific topical domain being searched. Second, among the stemmed preprocessors Farasa achieves the lowest latency in six of the seven categories (Culture: 10.0 ms, Finance: 7.2 ms, Politics: 5.8 ms, Religion: 2.8 ms, Sports: 3.4 ms, Technology: 3.2 ms), with Khoja marginally fastest in the remaining category (Medical: 4.5 ms versus Farasa’s 5.2 ms). Khoja’s narrow advantage there coincides with its smallest per-category result sets (125.5 documents per query, against its overall mean of 235.5), consistent with medical vocabulary producing less root-level conflation and therefore lower per-query transfer and decryption cost.

**Table 8:** Mean search latency by topical category (ms), 1,400 documents.

Category	NoPreprocessing	Khoja	ISRI	Lucene	Farasa
Culture	10.5	11.3	17.9	14.2	10.0
Finance	6.0	8.0	21.4	12.9	7.2
Medical	2.8	4.5	9.7	10.3	5.2
Politics	4.1	6.0	8.0	16.8	5.8
Religion	2.2	5.0	5.5	13.8	2.8
Sports	2.9	4.9	4.7	9.0	3.4
Technology	2.5	3.5	5.0	6.4	3.2

### 4.6. Qualitative analysis of preprocessing failure modes

Beyond aggregate metrics, we identify three qualitative failure modes that are informative for preprocessor selection in specific application domains as shown in Table 9.

**Table 9:** Qualitative failure cases across preprocessors.

Failure Mode	Query	Observation
Loanword failure	بيانات (data)	Khoja returns 0 results; root extraction fails on non-Arabic loanwords. ISRI: 425, Lucene: 84, Farasa: 175, NoPreprocessing: 28.
Over-stemming	صلاه (prayer)	ISRI returns 1 result; the stemmer over-reduces this term, collapsing it to a form matching very few indexed tokens. Khoja: 479, Lucene: 70, Farasa: 74, NoPreprocessing: 27.
Root conflation vs. surface matching	حاسوب (computer)	NoPreprocessing returns 1 result (exact token only). Lucene and Farasa return 4 results each — their conservative light stemming matches only close surface variants, preserving precision but limiting recall. ISRI returns 277 results via aggressive root conflation (حاسوب ‘computer’ → root حسب ‘calculate’), matching حساب (calculation), يحسب (he calculates), محسوب (calculated) and related forms — high recall but at the cost of semantic precision, as many matched tokens are conceptually distinct from “computer”. Khoja: 4 (root-based but conservative on this term).

The loanword failure of Khoja is a known limitation of root-based stemmers applied to Modern Arabic, which increasingly incorporates loanwords from English and French that do not conform to Semitic root-and-pattern morphology [7]. ISRI and Farasa are not affected by this failure mode for بيانات (data), returning 425 and 175 results respectively, compared to Khoja’s zero. In the Technology category, بيانات (data) is one such loanword, and Khoja returns zero results for this query while all other stemmed preprocessors return at least 84 results (ISRI: 425, Lucene: 84, Farasa: 175), compared to 28 for the normalization-only baseline.

The over-stemming of صلاه (prayer) by ISRI represents the opposite failure: the algorithm’s suffix-stripping rules collapse this word to a form that matches very few indexed tokens, resulting in near-zero retrieval breadth for a high-frequency religious term. Khoja retrieves substantially more documents for the same query (479 vs. ISRI’s 1). This illustrates the precision-recall trade-off inherent in different stemming paradigms and reinforces the importance of qualitative analysis alongside aggregate metrics. The third failure case — حاسوب (computer) — demonstrates this trade-off across all five configurations simultaneously. The normalization-only baseline and the light-stemming approaches (Lucene, Farasa) return only 1–4 results by matching near-surface forms only, whereas ISRI’s aggressive root conflation (حاسوب (computer) → root حسب (‘calculate’)) expands retrieval to 277 documents by matching semantically adjacent terms such as حساب (account/calculation) and محسوب (counted/considered). This root conflation inflates ISRI’s result sets without a corresponding gain in relevant material: its mean recall (0.277) remains below that of the normalization-only baseline (0.384) and of every other preprocessor, while its precision (0.556) is among the lowest, because the additional documents it returns are largely derivationally related rather than inflectionally relevant — topically adjacent, but not matching the query keyword or any inflectional variant of it under the relevance criterion of Section 3.6.

### 4.7. Search quality

**Fig 5:**
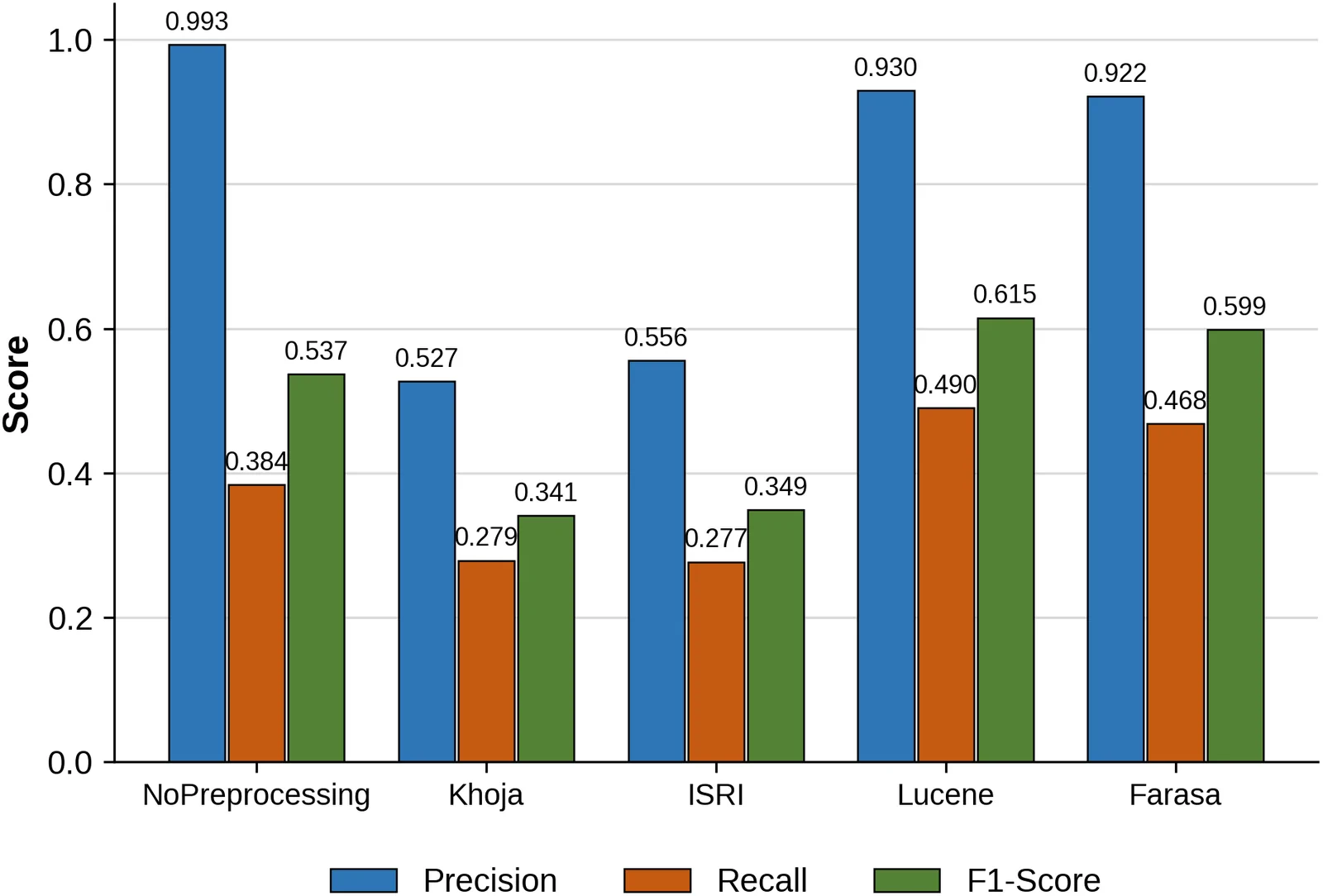
Search quality metrics by Arabic preprocessing pipeline. NoPreprocessing achieves near-perfect precision (0.993) but severely limited recall (0.384), while Lucene achieves the best F1 balance (0.615). Bars show mean precision, recall, and F1 values over 56 queries on the 1,400-document Khaleej corpus. F1 is computed per query as the harmonic mean of precision and recall and then averaged across queries.

Fig 5 illustrates the precision, recall, and F1 scores for each pipeline. The counterintuitive pattern is immediately visible: the normalization-only baseline achieves near-perfect precision (0.993) but severely limited recall (0.384), while Lucene achieves the best overall F1 balance (0.615). The annotation pool for search quality evaluation was constructed by depth-20 pooling across all five preprocessors: for each of the 56 benchmark queries, the top 20 documents returned by each preprocessor were merged into a single pool and duplicates were removed. This procedure yielded a total pool of 3,325 unique document–query pairs, of which 2,350 (70.7%) were judged relevant by the primary annotator. No query produced a pool with zero relevant documents, confirming that the benchmark queries are non-trivial and that at least one preprocessor retrieves relevant material for every query in the set.

Inter-annotator agreement was assessed on a stratified random subset of 665 document–query pairs (20.0% of the pool), with 95 pairs drawn from each of the seven topical categories. A second annotator applied the same token-level relevance rule described in Section 3.6 independently, without access to the primary annotator’s judgments. The two annotators agreed on 653 of 665 pairs (98.2% observed agreement), yielding Cohen’s κ = 0.956, which falls in the ‘almost perfect’ agreement range (κ ≥ 0.81) under the scale of Landis and Koch [31]. Agreement was uniformly high across all seven categories, with per-category κ values ranging from 0.894 (Culture) to 1.000 (Politics), and only 12 disagreements recorded across the entire subset. The high inter-annotator agreement confirms that the token-level relevance criterion is operationally unambiguous and that the primary annotator’s judgments constitute a reliable ground truth for the search quality evaluation.

Table 2 reports mean Precision, Recall, and F1-score for each preprocessor, averaged over all 56 queries. Relevance assessments are pool-based: documents not appearing in the pool for a given query are treated as not relevant, following standard pooling practice [30].

The normalization-only baseline achieves near-perfect mean precision (0.993), reflecting the fact that it retrieves very few documents per query (mean 47.2, Table 7) and those documents are nearly always relevant: they contain the exact query keyword token and satisfy the token-presence relevance criterion by definition. However, its mean recall is correspondingly low (0.384), confirming that the baseline fails to retrieve the majority of relevant documents in the pool due to its inability to match inflectional surface variants of the query keyword. For example, the query قرآن (‘Quran’) retrieves only 1 document under the baseline (precision = 1.000, recall = 0.036, F1 = 0.069), against a pool of 28 relevant documents, because the baseline indexes only the exact normalized surface form and misses documents containing inflectional variants.

Root-based stemmers exhibit the opposite pattern. Khoja and ISRI achieve the lowest mean precision of all five configurations (0.527 and 0.556, respectively), confirming that their broad retrieval breadth — documented in Section 4.4 — reflects systematic derivational over-conflation rather than genuine recall improvement. Despite retrieving a mean of 235.5 documents per query (Table 7), Khoja achieves a mean recall of only 0.279, lower than the normalization-only baseline (0.384). This counter-intuitive result arises because root-based conflation causes Khoja to retrieve large numbers of documents containing derivationally related but lexically distinct forms of the query keyword — forms that are judged not relevant under the token-level criterion — while simultaneously failing to index some surface forms correctly. The loanword failure documented in Section 4.6 is a direct illustration of this phenomenon: Khoja retrieves zero documents for the query بيانات (‘data’), yielding precision = 0.000, recall = 0.000, and F1 = 0.000, because root extraction fails entirely on this non-Semitic loanword. ISRI exhibits an analogous failure for صلاه (‘prayer’), retrieving only 1 document (precision = 1.000, recall = 0.024, F1 = 0.047) due to aggressive over-reduction. The resulting F1 scores for Khoja (0.341) and ISRI (0.349) are the lowest among all five configurations, establishing that root-based approaches incur a substantial quality cost relative to their efficiency advantage.

Lucene achieves the highest mean F1-score (0.615) and the highest mean recall (0.490) of all five preprocessors, while maintaining high precision (0.930) comparable to the normalization-only baseline. This result is consistent with the design of the light stemming algorithm underlying the Lucene Arabic Analyzer [6]: by normalizing inflectional suffixes without aggressive derivational conflation, Lucene retrieves a broader set of genuinely relevant documents while avoiding the false positives that penalize root-based approaches. Farasa achieves the second-highest F1 (0.599) with precision (0.922) and recall (0.468) closely comparable to Lucene. The Lucene–Farasa F1 gap (0.016) is small, and both substantially outperform the root-based stemmers (F1 advantage over Khoja: + 0.274 for Lucene, + 0.258 for Farasa).

Table 10 reports mean F1-score by topical category. The highest F1 scores across stemmed preprocessors are observed in Medical (Farasa F1 = 0.727, Lucene = 0.634) and Technology (Lucene = 0.657, Farasa = 0.616). The lowest F1 scores are observed under the root-based stemmers in the Finance category (Khoja: 0.203, ISRI: 0.289), consistent with the loanword failure mode documented in Section 4.6: the Finance query set includes the loanword بنك (‘bank’), on which Khoja’s root extraction fails (F1 = 0.028). Among stemmed preprocessors, Lucene achieves the highest F1 in five of the seven categories (Finance, Politics, Religion, Sports, Technology); Farasa achieves the highest F1 among stemmed preprocessors in Culture (0.607) and Medical (0.727). In Sports, the normalization-only baseline achieves the highest overall F1 (0.622), suggesting that exact-token matching is particularly effective for Sports vocabulary in this corpus.

**Table 10:** Mean F1-score by topical category per preprocessor (1,400 documents, 8 queries per category). Bold values indicate the highest F1 in each row.

Category	NoPreprocessing	Khoja	ISRI	Lucene	Farasa
Culture	0.554	0.397	0.355	0.551	**0.607**
Finance	0.544	0.203	0.289	**0.598**	0.589
Medical	0.552	0.336	0.485	0.634	**0.727**
Politics	0.448	0.357	0.413	**0.579**	0.542
Religion	0.492	0.351	0.310	**0.684**	0.620
Sports	**0.622**	0.314	0.301	0.605	0.490
Technology	0.546	0.429	0.292	**0.657**	0.616

**Statistical significance of quality differences.** Differences in search quality were tested for statistical significance using the same Wilcoxon signed-rank procedure applied to latency (Section 3.5), on per-query F1-scores across all ten preprocessor pairs (n = 56 paired observations per comparison; full results in S2 Appendix). Six of the ten differences are significant at the Bonferroni-corrected threshold (p < 0.005). Both Lucene and Farasa significantly outperform both root-based stemmers (all four comparisons p < 0.0001), confirming that the quality advantage of light stemming and segmentation over root extraction is not an artifact of sampling. Three differences are not significant: Lucene versus Farasa (W = 122.0, p = 0.424), Khoja versus ISRI (W = 562.0, p = 0.790), and the normalization-only baseline versus Farasa (W = 174.0, p = 0.092); the baseline versus Lucene comparison (W = 138.0, p = 0.031) does not survive Bonferroni correction. The Lucene–Farasa result is consequential for preprocessor selection: although Lucene attains the highest mean F1, its advantage over Farasa lies within sampling variation, so the two are statistically indistinguishable in retrieval quality on this corpus, and Farasa’s substantially lower latency is not purchased at a measurable quality cost.

Taken together, the search quality results establish three conclusions that complement the latency findings of Sections 4.2–4.3. First, morphological preprocessing does not uniformly improve search quality: root-based stemmers (Khoja, ISRI) degrade precision substantially relative to the normalization-only baseline, and their recall advantage is negligible or negative under a token-level relevance criterion. Second, light stemming (Lucene) and morphological segmentation (Farasa) achieve the best quality-efficiency trade-off: both maintain high precision (≥0.922) while improving recall over the baseline by 10.6 percentage points (Lucene: 0.490 vs. 0.384) and 8.4 percentage points (Farasa: 0.468 vs. 0.384). Third, addressing RQ3, the appropriate preprocessor choice depends on the application’s quality-efficiency requirements: Farasa offers the best latency with near-Lucene quality (F1 = 0.599), while Lucene offers the highest mean F1 (0.615) at the cost of slightly slower search performance, although the Lucene–Farasa quality difference is not statistically significant. These findings are discussed further in Section 5.

### 4.8. Scalability analysis

To evaluate the scalability of Arabic DSSE beyond the annotated 1,400-document corpus, and to assess performance under a realistic deployment configuration rather than the consumer hardware used for the quality evaluation, we conducted an extended evaluation on AWS EC2 cloud infrastructure with the client and server deployed as separate processes, following the scalability protocol described in Section 3.4 and using the 400-query pool described in Section 3.3. All five preprocessing configurations — including the normalization-only baseline — were evaluated across five corpus sizes (1,000; 5,000; 10,000; 25,000; and 45,500 documents), spanning the full Khaleej corpus, using the corrected bit-packed index implementation (Section 3.4). This experiment directly addresses whether the preprocessing performance characteristics observed at 1,400 documents persist at deployment scale.

**Fig 6:**
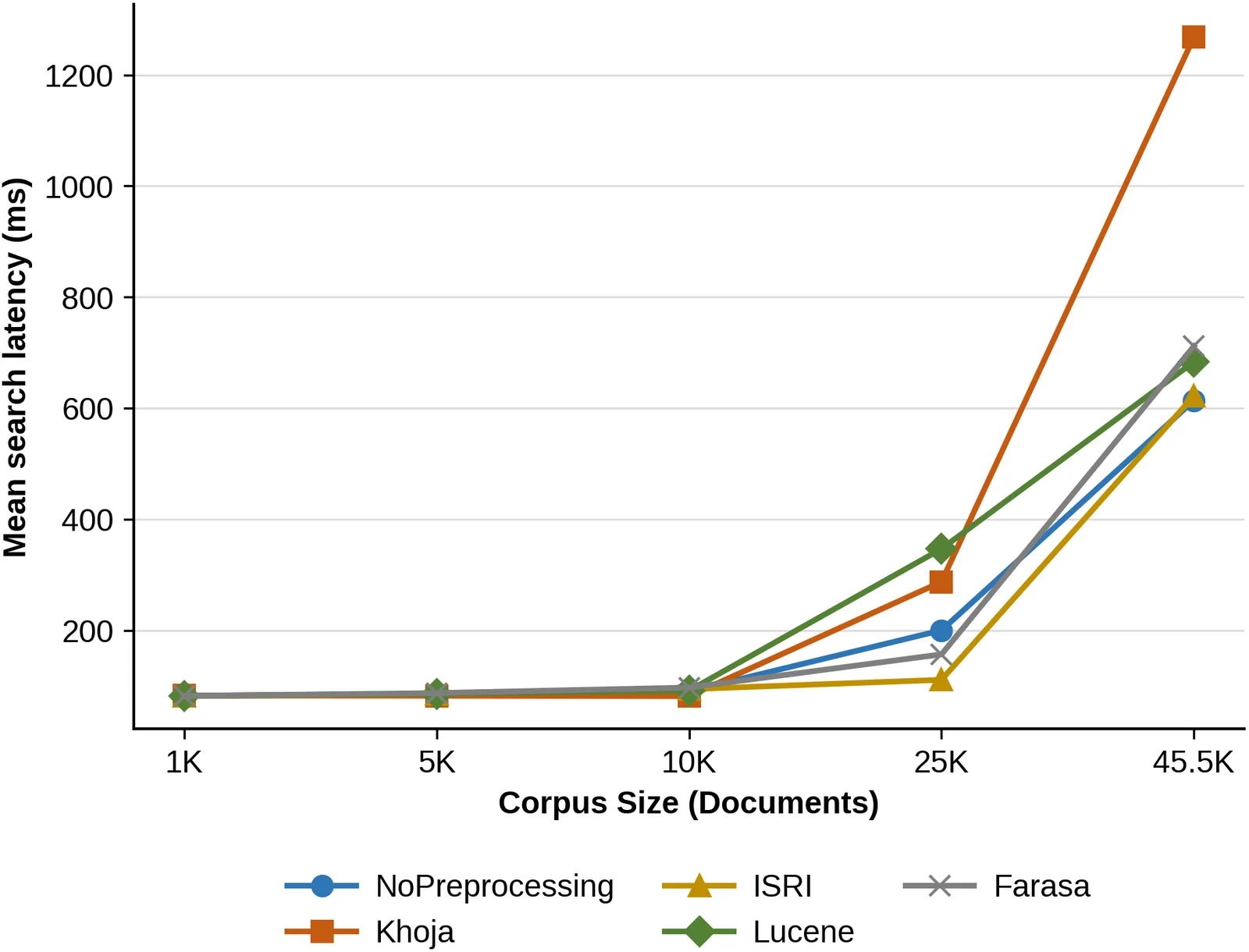
Mean search latency versus corpus size for all five preprocessors (1,000–45,500 documents, RTT = 0).

Table 11 and Fig 6 report mean search latency across the five corpus sizes. Two findings emerge. First, all five configurations — including the normalization-only baseline — operate successfully at 45,500 documents, confirming that the memory limitation reported for the earlier per-entry implementation was an artifact of the data structure rather than an intrinsic property of the scheme or of unprocessed Arabic. Second, latency grows sub-linearly with corpus size for all preprocessors up to approximately 10,000 documents, then rises steeply. At smaller corpus sizes (1,000–10,000 documents), mean latencies for all five configurations remain within a narrow band (approximately 82–98 ms; Table 11): per-query cost in this regime is dominated by fixed protocol and row-processing overhead rather than by result-set size, so Khoja’s larger result sets incur no visible penalty. Beyond 10,000 documents, result handling becomes the dominant cost, producing the divergence visible in Fig 6. At 45,500 documents, Khoja exhibits the highest latency (1,267.9 ms) despite having the smallest vocabulary, because its aggressive root conflation returns the largest result sets (12,720 documents per query on average). The normalization-only baseline remains comparatively efficient (614.3 ms) because it matches the fewest documents (4,104 per query).

**Table 11:** Mean search latency (ms) by corpus size and preprocessor, RTT = 0.

Corpus size	NoPreprocessing	Khoja	ISRI	Lucene	Farasa
**1,000**	82.5	83.4	82.9	83.0	82.8
**5,000**	86.1	83.0	86.6	86.5	87.9
**10,000**	90.9	82.3	94.9	92.2	97.8
**25,000**	200.8	288.0	111.7	347.3	157.5
**45,500**	614.3	1267.9	622.6	683.9	711.8

**Fig 7:**
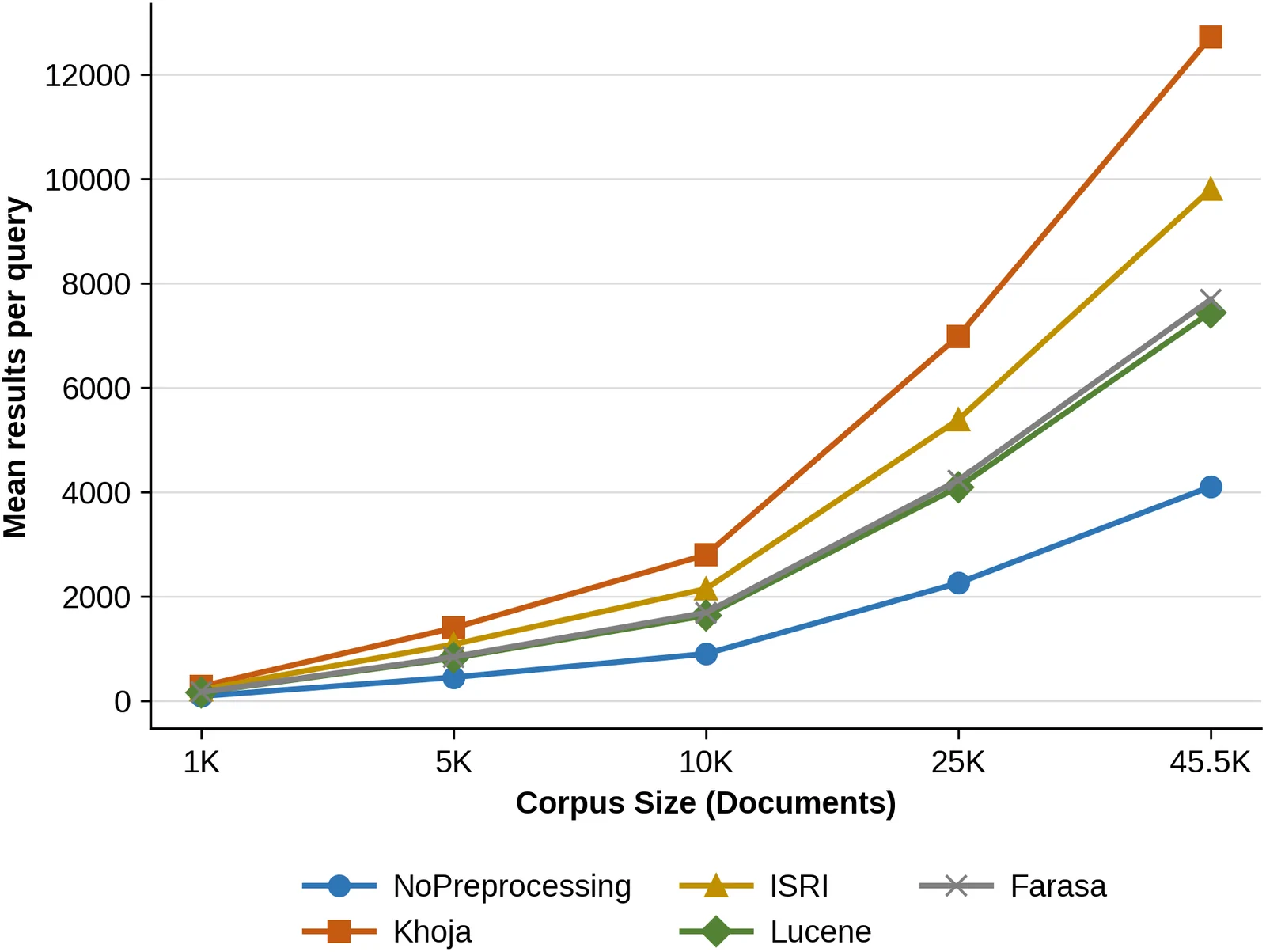
Mean results per query versus corpus size, showing that retrieval breadth scales approximately linearly and drives the latency growth in Fig 6.

Table 12 and Fig 7 report retrieval breadth across corpus sizes and explain the latency behavior in Table 11 and Fig 6. Result-set size scales approximately linearly with corpus size for every preprocessor, and the ordering is stable: root-based stemmers (Khoja, ISRI) consistently return the most documents per query, while the normalization-only baseline returns the fewest. This is consistent at scale with the mechanism identified at 1,400 documents: beyond the fixed-overhead regime, differences in per-query latency among preprocessors are driven primarily by result-set size, most starkly for Khoja. The practical implication for the selection framework is that aggressive root conflation, while maximizing retrieval breadth, can impose severe latency growth as corpora scale — Khoja’s mean latency grows 15× from 1,000–45,500 documents, versus 7–9× for every other configuration — whereas light stemming (Lucene) and segmentation (Farasa) combine moderate latency scaling with superior retrieval quality.

**Table 12:** Mean results per query (retrieval breadth) by corpus size and preprocessor.

Corpus size	NoPreprocessing	Khoja	ISRI	Lucene	Farasa
**1,000**	90	279	216	163	168
**5,000**	451	1398	1083	819	843
**10,000**	901	2798	2148	1636	1691
**25,000**	2258	6989	5390	4096	4228
**45,500**	4104	12720	9803	7445	7691

#### 4.8.1. Memory profile.

The memory footprint of the encrypted index follows directly from the bit-packed representation: for a vocabulary of m keywords and a corpus of n documents, the index requires 2 × m × n bits (one bit for the value plane and one for the state plane per cell). At 1,400 documents the normalization-only baseline (m = 76,036) requires approximately 2 × 76,036 × 1,400/ 8 ≈ 26.6 MB, versus 4.6 MB for Khoja (m = 13,010) — an 83% reduction proportional to the vocabulary reduction. At 45,500 documents the baseline index reaches approximately 2 × 479,703 × 45,500/ 8 ≈ 5.5 GB, which fits comfortably within the 24 GB server allocation. This analytical model is consistent with the measured heap usage observed during the scalability experiments and confirms that vocabulary size is the primary determinant of encrypted-index memory, with preprocessing yielding proportional memory savings.

Preprocessing time is reported separately from search latency throughout this study. The client-side token-generation time — the cost of preprocessing a query and deriving its search token — was measured at under 0.1 ms per query for all five configurations at every corpus size (mean 0.02–0.07 ms), and is therefore negligible relative to the encrypted search operation. Index construction (one-time preprocessing of the full corpus) is a setup cost incurred once per deployment rather than per query; it scales with corpus size and vocabulary but does not affect the per-query search latency reported in Tables 10 and 11.

## 5. Discussion

### 5.1. The role of preprocessing in scalable encrypted search

**Fig 8:**
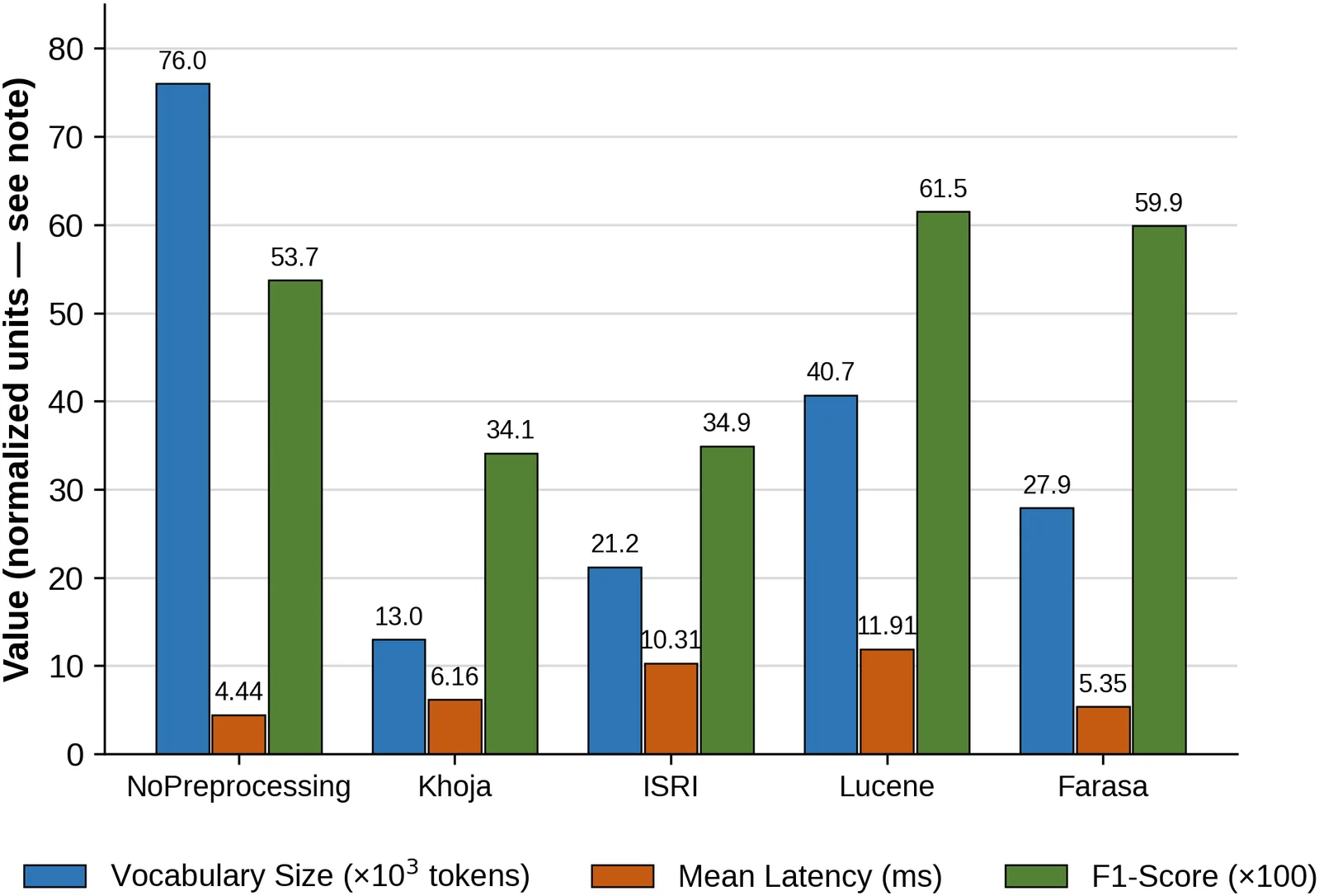
Summary of preprocessing pipeline performance across three evaluation dimensions. Vocabulary size (×10³ tokens), mean end-to-end search latency (ms), and F1 score (scaled by 100 for comparability) are shown for all five pipelines. All values are means over 56 benchmark queries on the 1,400-document Khaleej corpus. F1 is computed per query as the harmonic mean of precision and recall and then averaged across queries.

Fig 8 summarizes the performance of all five preprocessing pipelines across the three key dimensions evaluated in this paper: vocabulary size, mean search latency, and F1 score. Our results demonstrate that the primary practical impact of Arabic morphological preprocessing in the encrypted search context lies in retrieval quality and index size rather than in per-query latency. Vocabulary reduction of up to 83% translates directly into proportionally smaller encrypted indexes and lower transmission cost: the normalization-only baseline produces an index of 76,036 keyword rows, whereas Khoja reduces this to 13,010. At scale this difference is substantial — our cloud-based scalability experiments (Section 4.8) show that index construction and transmission cost grow with vocabulary size across corpora up to 45,500 documents. While an earlier implementation of our index encountered a memory limitation on consumer hardware, we found this to be an artifact of the index data structure rather than an intrinsic property of the scheme; with a bit-packed index representation (Section 3.4), all five configurations — including the normalization-only baseline — operate on corpora up to 45,500 documents. The enduring benefit of preprocessing is therefore improved retrieval quality and reduced index footprint, not latency feasibility. This nuances RQ1: aggressive stemming is not required for latency-feasible encrypted search — the normalization-only baseline is in fact fastest per query — but vocabulary reduction remains highly valuable for controlling index size and transmission cost, and light stemming or segmentation is preferred for its superior retrieval quality (Section 4.7).

The vocabulary reduction achieved by stemming directly translates to a smaller encrypted index, faster index construction, and lower search latency. Khoja’s 83% vocabulary reduction relative to the baseline produces the lightest encrypted index and the highest retrieval breadth per query (4.99 × the baseline). However, retrieval breadth alone is not evidence of retrieval quality: root-based conflation means Khoja systematically retrieves documents containing derivationally related forms that do not contain the original query keyword, which under the preprocessor-neutral relevance criterion constitute false positives. The search quality results (Table 2) quantify this cost: Khoja’s precision falls to 0.527 — a 47.0% drop from the baseline’s 0.993 — and its precision of 0.527 is the lowest of all five configurations. For deployments where retrieval coverage is the primary concern — such as legal discovery or medical record search — Khoja provides the most favorable efficiency-coverage trade-off subject to its known loanword limitation, but practitioners should be aware that its high retrieval breadth partially reflects over-conflation rather than genuine recall.

### 5.2. The latency-breadth trade-off

Farasa achieves the second-fastest search latency (5.35 ms) and a moderate retrieval breadth (99.3 documents per query) among the stemmed preprocessors. The mechanistic explanation lies in Farasa’s segmentation approach: rather than conflating words to a common root, Farasa preserves stem morphemes with greater surface specificity, producing a more discriminative index vocabulary (27,880 tokens vs. 13,010 for Khoja). More discriminative tokens match fewer documents per query, so the server returns a smaller encrypted result set. Since IM-DSSE search decrypts an entire index row and transmits all matching document identifiers, a smaller result set means less data transmitted and less decryption work, directly reducing end-to-end latency. This is consistent with Farasa’s design philosophy: it performs morphological segmentation rather than root extraction, preserving more of the word’s surface structure. The resulting index tokens are more discriminative, leading to fewer but more precise matches. Lucene achieves low latency variance (SD = 7.29 ms) relative to the root-based stemmers for a related but distinct reason: its light stemming produces a vocabulary of intermediate size (40,746 tokens) with more uniform document frequency distribution across query terms. Root-based stemmers produce highly skewed distributions — some roots match hundreds of documents while others match very few — causing high variance in per-query result set size and therefore high variance in latency. Lucene’s light stemmer avoids this skew by preserving more lexical distinctions, yielding consistent result set sizes and predictable latency across diverse query types.

Khoja and ISRI occupy the opposite end of this trade-off, with the highest retrieval breadth (235.5 and 223.5 respectively) at the cost of higher latency. The performance ordering in ascending mean search latency — NoPreprocessing (4.44 ms), Farasa (5.35 ms), Khoja (6.16 ms), ISRI (10.31 ms), Lucene (11.91 ms) — reflects a consistent pattern: more aggressive normalization produces a smaller, faster-to-search index but with broader, less discriminative retrieval. Under the preprocessor-neutral relevance criterion adopted in this study, documents retrieved through derivational over-conflation — for example, documents about شاعر (poet) retrieved for a query on شعر (poetry) — constitute false positives that reduce precision. The search quality results (Table 2) confirm and quantify this pattern. Khoja and ISRI achieve the lowest precision scores (0.527 and 0.556, respectively) and the lowest F1 scores (0.341 and 0.349) of all five configurations. Despite retrieving far more documents than the normalization-only baseline, both root-based stemmers achieve lower recall (Khoja 0.279, ISRI 0.277) than the baseline (0.384), because derivational over-conflation causes them to retrieve large numbers of irrelevant documents while simultaneously failing to correctly index some query-relevant surface forms. The data therefore establish that for Arabic DSSE under an inflectional relevance criterion, root-based stemming produces neither the best precision nor the best recall: it sacrifices precision through over-conflation without achieving commensurate recall gains. Addressing RQ2 and RQ4: Farasa provides the optimal latency-breadth balance, while Khoja and ISRI maximize breadth at a substantial precision cost.

### 5.3. Lucene latency performance

Although Lucene records the highest mean latency among the five configurations (11.91 ms), it exhibits the lowest variability relative to its mean of the five configurations (SD = 7.29 ms) and a stable median (11.00 ms), indicating consistent and predictable search performance relative to its typical latency across diverse query types. This consistency arises because Lucene’s light stemming produces a more uniform document-frequency distribution than root-based approaches, avoiding the highly skewed result-set sizes that cause Khoja and ISRI to spike in latency for high-frequency roots. The pairwise comparisons (S2 Appendix) confirm that Lucene is significantly slower than Farasa (p < 0.001), reflecting its larger mean result set; however, for deployments where latency predictability matters more than absolute speed, Lucene’s low variance is advantageous. Critically, Lucene also achieves the highest retrieval quality (F1 = 0.615, Section 4.7), making it the preferred choice where search quality is the primary objective.

### 5.4. A preprocessor selection framework for Arabic DSSE

**Scope of the framework.** The framework proposed here has two layers, and they generalize differently. The first layer is structural: a set of decision axes — index compactness and memory ceiling, per-query latency, retrieval breadth, and retrieval quality — together with the mechanisms that connect each axis to the preprocessing choice. These mechanisms follow from the architecture of matrix-based DSSE rather than from properties of this particular corpus. Vocabulary size determines the number of rows in the encrypted index and therefore its memory footprint; the number of documents matched by a query is the principal driver of the volume of encrypted data transferred and decrypted, and therefore of per-query latency once the fixed protocol overhead is exceeded (Section 4.8), although per-query token generation contributes an additional, preprocessor-dependent cost (Section 5.3); and derivational conflation systematically trades precision for retrieval breadth under an inflectional relevance criterion (Section 4.7). These relationships should hold for any DSSE construction whose index size scales with vocabulary and whose search cost scales with result-set size, and they yield falsifiable predictions for other settings — for example, that root extraction will yield the smallest encrypted index and the largest result sets in any such scheme.

The second layer is quantitative: the specific vocabulary reductions, latencies, and F1-scores reported in Section 4, and the thresholds derived from them. These are calibrated to Gulf Arabic news text, the IM-DSSE construction, and the protocol described in Section 3, and are properly read as parameters of this setting rather than as universal constants. A different register, dialect, or domain may shift vocabulary statistics and therefore the magnitude — though, by the structural argument above, not the direction — of these effects; a construction with a different index representation may weaken the vocabulary–memory coupling on which the index-compactness guideline rests. Re-instantiating the framework in a new setting therefore requires re-estimating the parameters rather than re-deriving the structure, and the deposited benchmark query set, harness, and analysis scripts are provided so that this re-estimation can be carried out directly. Validation across additional corpora and DSSE constructions is the principal direction for future work (Section 5.5).

Based on our findings across all four research dimensions (RQ1–RQ4), we propose the following evidence-based guidelines for practitioners deploying Arabic DSSE systems. Each guideline maps directly to the empirical results reported in Section 4:

- Morphological preprocessing is strongly recommended for Arabic DSSE wherever retrieval quality or index compactness matters. While the normalization-only baseline achieves the lowest per-query latency (because it matches the fewest documents) and operates at full corpus scale, it does so at the cost of the largest encrypted index and the poorest retrieval recall; light stemming and segmentation are therefore preferable in practice. These recommendations rest on differences that are statistically significant: the pairwise latency differences among preprocessors (p < 0.05, most p < 0.001); the precision advantage of the normalization-only baseline over both root-based stemmers (p < 0.001); the F1 advantage of both Lucene and Farasa over both root-based stemmers (p < 0.001); and Lucene’s recall advantage over the baseline (p = 0.005). The F1 differences between the baseline and the light-stemming and segmentation pipelines are smaller and do not survive Bonferroni correction (Section 4.7); the recommendation to prefer the latter therefore rests on their recall and index-compactness advantages rather than on aggregate F1 alone.
- For latency-critical applications requiring predictable real-time search, Farasa offers a very low mean search latency (5.35 ms) and low median (4.00 ms) among the quality-competitive preprocessors, making it an attractive choice for latency-sensitive production deployment. (The normalization-only baseline is nominally faster still, at 4.44 ms, but its poor recall — see Section 4.7 — makes it unsuitable where retrieval quality matters.) Note that Lucene achieves the lowest variance relative to its mean of the five configurations (SD = 7.29 ms; coefficient of variation 0.61) and therefore the most consistent latency relative to its typical response time.
- For breadth-critical applications requiring maximum document coverage (document discovery, legal search, medical record retrieval), Khoja or ISRI maximize retrieval breadth, recovering approximately 5 × more documents per query than the baseline. However, practitioners should note that a portion of this breadth reflects derivational over-conflation rather than genuine recall: root-based stemmers will retrieve documents containing related but lexically distinct forms of the query keyword. The search quality evaluation (Table 2) confirms this concern: Khoja precision = 0.527, ISRI precision = 0.556, versus 0.993 for the baseline — a drop of approximately 47% and 44% respectively for the two root-based stemmers. ISRI is preferred over Khoja in corpora with significant non-Arabic vocabulary due to Khoja’s loanword failure.
- For corpora with substantial loanword content (technical, scientific, or mixed-language documents), Khoja should be avoided due to its root extraction failure on non-Semitic vocabulary. ISRI or Farasa are more robust in this context.
- Based on the search quality results (Table 2), Lucene and Farasa are recommended for general-purpose Arabic DSSE deployment: both achieve precision above 0.92 and F1 above 0.59, with Farasa offering the additional advantage of very low search latency (5.35 ms), and Lucene offering the lowest variance relative to its mean (SD = 7.29 ms) for the most predictable performance relative to its typical latency.

Table 13 summarizes the advantages and limitations of each preprocessing method observed across the four evaluation dimensions, consolidating the evidence underlying the guidelines above.

**Table 13:** Advantages and limitations of each preprocessing method, as observed in this evaluation (1,400-document Khaleej corpus; scalability figures from Section 4.8). Values are drawn from Tables 2, Tables 4–12.

Stage/ method	Advantages	Limitations
Character normalization only (NoPreprocessing, baseline)	Highest precision (0.993); lowest mean latency (4.44 ms) and decryption cost (2.29 ms); deterministic and dependency-free; smallest per-query result sets (47.2 documents).	Largest encrypted index (76,036 unique tokens); lowest recall (0.384); no matching of inflected variants, so morphologically related documents are missed (e.g., حاسوب returns 1 document).
Light stemming (Lucene Arabic Analyzer)	Highest mean F1 (0.615) and recall (0.490) with near-baseline precision (0.930); 46.4% vocabulary reduction; most predictable latency (lowest coefficient of variation).	Highest mean latency of the five configurations (11.91 ms); smallest vocabulary reduction among the stemmed pipelines; quality advantage over Farasa is not statistically significant (p = 0.424).
Morphological segmentation (Farasa)	Lowest latency among quality-competitive pipelines (5.35 ms) with low decryption cost (3.82 ms); F1 (0.599) statistically indistinguishable from Lucene; 63.3% vocabulary reduction.	Retrieval breadth (99.3 documents per query) is the narrowest of the stemmed pipelines, which limits coverage in recall-oriented deployments; F1 falls to 0.000 on one benchmark query (بطوله).
Root extraction, dictionary-assisted (Khoja)	Greatest vocabulary reduction (82.9%, to 13,010 tokens) and greatest retrieval breadth (235.5 documents per query); low mean latency at small scale (6.16 ms).	Lowest precision (0.527, a 47% reduction) and lowest F1 (0.341); fails on loanwords (بيانات returns 0 documents); steepest latency growth with corpus size (15× versus 7–9×); requires external lookup tables.
Root extraction, dictionary-free (ISRI)	Large vocabulary reduction (72.2%) and broad retrieval (223.5 documents per query) with no dictionary dependency.	Low precision (0.556) and F1 (0.349); over-conflation collapses distinct forms (صلاه returns 1 document); high decryption cost (8.07 ms); result counts for four queries vary between runs (Section 3.4).

### 5.5. Limitations

Several limitations of this study should be acknowledged. First, absolute latency values are specific to the hardware used — an Apple M1 machine for the 1,400-document quality evaluation and an AWS EC2 r6i.2xlarge instance for the scalability evaluation — and different JVM implementations or hardware specifications may yield different absolute timings; the relative ordering among preprocessors, rather than the absolute values, is the reproducible finding. In addition, the scalability experiments were conducted over a loopback interface with negligible network round-trip time (RTT = 0) in order to isolate computational scaling; deployments with non-trivial client–server network latency would add a preprocessor-independent constant to end-to-end response time, and evaluating that regime is left to future work. Second, the Khaleej corpus comprises Gulf Arabic news text, which may not generalize to other Arabic registers, dialects, or domain-specific corpora such as legal, medical, or social media text. Different domains may exhibit different vocabulary size and morphological complexity characteristics, which could alter the relative performance ordering of preprocessors. Third, the benchmark query set of 56 keywords, while meeting TREC-2002 standards, is necessarily smaller than larger TREC benchmarks; more queries would provide more stable aggregate estimates, particularly for mean Precision and F1-score. Fourth, inter-annotator agreement was assessed on a 20% stratified subset (665 pairs, κ = 0.956); full dual annotation of the complete pool was not performed and remains a direction for future work to further strengthen the credibility of the relevance judgments. Fifth, this study evaluates preprocessing within the IM-DSSE scheme only; different DSSE constructions with different index structures (e.g., inverted index-based schemes, forward-private constructions) may exhibit different sensitivity to vocabulary size and may therefore respond differently to preprocessing choices. Sixth, the evaluation is limited to single-keyword queries. This is a property of the underlying cryptographic construction rather than of the evaluation design: IM-DSSE, like the foundational SSE and DSSE schemes it follows, defines search as single-keyword trapdoor matching — the server returns the set of matching encrypted document identifiers and performs no filtering, sorting, or ranking, so there is no server-side complex-query machinery to evaluate. Single-keyword evaluation also isolates the experimental variable of interest, since Arabic morphological preprocessing operates at the level of individual tokens; multi-keyword queries would confound preprocessing effects with intersection-strategy effects. We note, however, that our central finding permits a prediction for conjunctive queries: a k-keyword conjunction implemented over IM-DSSE (k trapdoors issued, identifier sets intersected client-side) costs the sum of k single-keyword searches plus an intersection whose cost is linear in the retrieved set sizes. Because per-query latency is driven primarily by result-set size, the preprocessor ordering observed here is expected to persist — and the disadvantage of root-based stemmers to sharpen — under conjunction, as their inflated result sets would increase both the per-keyword and the intersection cost. Native phrase and conjunctive search require different cryptographic constructions with different index structures and leakage profiles; empirically validating this prediction within such schemes is an important direction for future work. Seventh, the framework presented in this study is grounded in Arabic morphological preprocessing; however, the underlying principles are likely to generalize to other morphologically rich languages that share similar characteristics. Persian (Farsi), for example, shares Arabic’s right-to-left script and many loanwords, and several of the tools evaluated here have Persian variants. Hebrew similarly exhibits root-and-pattern morphology with cliticization, making vocabulary explosion a likely challenge in Hebrew DSSE. The framework’s core insight — that more aggressive normalization reduces vocabulary size and latency at the cost of retrieval precision — is expected to hold for any language where morphological variation creates index vocabulary inflation. Empirical validation of this generalization for Persian, Hebrew, Turkish, and other morphologically complex languages represents an important direction for future work.

## 6. Conclusion

This paper has presented the first empirically grounded preprocessor selection framework for Arabic Dynamic Searchable Symmetric Encryption, built on a systematic evaluation of preprocessing strategies within the IM-DSSE scheme of Hoang et al. [3]. Using the Khaleej Arabic news corpus and a 56-query benchmark across seven topical categories, we evaluated four stemmed preprocessors against a normalization-only baseline under a preprocessor-neutral, inflectional-level relevance criterion.

Our principal findings are as follows. First, morphological preprocessing reduces Arabic keyword vocabulary by 46–83% relative to the normalization-only baseline, proportionally reducing encrypted-index size and transmission cost; scalability experiments confirm all five configurations operate on corpora up to 45,500 documents on cloud infrastructure. Second, per-query search latency is driven primarily by result-set size rather than vocabulary size: the normalization-only baseline is fastest (4.44 ms) because it matches the fewest documents, while among quality-competitive preprocessors Farasa achieves a very low mean latency (5.35 ms) alongside the second-highest F1 (0.599), making it the best-balanced configuration where both retrieval quality and latency matter: its F1 is statistically indistinguishable from Lucene’s while its mean latency is less than half. Third, Lucene achieves the highest mean F1 (0.615) and recall (0.490) with near-baseline precision (0.930), confirming that light stemming provides an excellent precision–recall balance under the inflectional relevance criterion; its advantage over Farasa on these measures is not statistically significant (Section 4.7), so the two are best regarded as jointly preferred where retrieval quality is the priority. Fourth, root-based stemmers (Khoja F1 = 0.341, ISRI F1 = 0.349) achieve the lowest search quality of all five configurations despite their greater retrieval breadth: their high false-positive rate from derivational over-conflation reduces precision by approximately 47% (Khoja) and 44% (ISRI) relative to the baseline without achieving commensurate recall gains. Fifth, retrieval breadth differs substantially across preprocessors: root-based stemmers (Khoja 235.5, ISRI 223.5 documents per query) recover approximately 5 × more documents per query than the baseline (47.2), but search quality results confirm that a substantial portion of this breadth reflects derivational over-conflation rather than genuine recall improvement. Sixth, Lucene records the highest mean latency among the five configurations (11.91 ms) but the lowest variance relative to its mean among the five configurations (SD = 7.29 ms; coefficient of variation 0.61) and a stable median (11.00 ms), making it the most consistent performer relative to its typical latency; its light stemming avoids the skewed result-set sizes that cause root-based stemmers to spike on high-frequency roots. Seventh, qualitative per-keyword analysis reveals Khoja’s complete failure on Arabic loanwords (F1 = 0.000 for بيانات ‘data’, F1 = 0.028 for بنك ‘bank’), ISRI’s failure on زكاه (‘zakat’, Islamic almsgiving; F1 = 0.000) and over-reduction of صلاه (‘prayer’; F1 = 0.047), and Farasa’s failure on بطوله (‘championship’; F1 = 0.000) — illustrating that no single preprocessor dominates across all query types.

These findings provide concrete, evidence-based guidance for practitioners deploying Arabic DSSE systems: preprocessing is not required for the system to scale — all five configurations operate at full corpus size — but it is what makes encrypted Arabic search compact and accurate, and the choice of preprocessor should be guided by application requirements: Farasa is recommended as the default choice, offering very low latency (5.35 ms) with competitive search quality (F1 = 0.599, precision = 0.922); Lucene is preferred where search quality is the primary concern (F1 = 0.615, recall = 0.490); Khoja or ISRI are suitable where maximum retrieval breadth is required (approximately 5 × the baseline), with the caveat that precision drops approximately 44–47% due to derivational over-conflation; root-based stemmers should be avoided where precision is important. Because the Lucene–Farasa difference in mean F1 is not statistically significant (p = 0.424; Section 4.7), the choice between them may be governed by latency and predictability rather than by retrieval quality alone.

## Supporting information

S1 Appendix Benchmark Query Set. The complete set of 56 Arabic benchmark queries used in the evaluation, distributed across seven topical categories of the Khaleej corpus, with English glosses.
(DOCX)

S2 Appendix Pairwise Statistical Comparisons. Pairwise Wilcoxon signed-rank test results for search latency among the four stemmed preprocessors (Table B.1) and for search quality (per-query F1) among all five configurations (Table B.2). Based on 1,400 documents, 56 queries, and 5 runs per query.
(DOCX)

S1 File Scalability query pool. The complete pool of 400 Arabic queries used in the scalability evaluation (Section 4.8), comprising the 56 curated benchmark keywords (S1 Appendix) followed by 344 high-frequency Khaleej corpus words. UTF-8 plain text, one query per line.
(TXT)
