# Supplementary material for: Arabic text preprocessing for dynamic searchable symmetric encryption: An empirical evaluation and preprocessor selection framework
Source: PLoS One. 2026 Sep 21;21(9):e0351281. doi: 10.1371/journal.pone.0351281 (PMC13592697; doi:10.1371/journal.pone.0351281)
Supplement: Supplementary file 1 [file pone.0351281.s001.docx]

**S1 Appendix. Benchmark Query Set**

*Arabic Text Preprocessing for Dynamic Searchable Symmetric Encryption: An Empirical Evaluation and Preprocessor Selection Framework*

This appendix lists the complete set of 56 Arabic benchmark queries used in the evaluation, distributed across the seven topical categories of the Khaleej corpus (eight queries per category), with English glosses.

| **#** | **Category** | **Query (normalized Arabic)** | **English gloss** |
| --- | --- | --- | --- |
| 1 | Culture | معرض | exhibition |
| 2 | Culture | مهرجان | festival |
| 3 | Culture | فنون | arts |
| 4 | Culture | مسرح | theater |
| 5 | Culture | روايه | novel |
| 6 | Culture | ادب | literature |
| 7 | Culture | شعر | poetry |
| 8 | Culture | ثقافه | culture |
| 9 | Finance | شركه | company |
| 10 | Finance | استثمار | investment |
| 11 | Finance | بنك | bank |
| 12 | Finance | اقتصاد | economy |
| 13 | Finance | تجاره | trade |
| 14 | Finance | ميزانيه | budget |
| 15 | Finance | سوق | market |
| 16 | Finance | اسهم | shares |
| 17 | Medical | صحه | health |
| 18 | Medical | علاج | treatment |
| 19 | Medical | مستشفى | hospital |
| 20 | Medical | مريض | patient |
| 21 | Medical | وباء | epidemic |
| 22 | Medical | دواء | medicine |
| 23 | Medical | جراحه | surgery |
| 24 | Medical | طبيب | physician |
| 25 | Politics | رئيس | president |
| 26 | Politics | انتخاب | election |
| 27 | Politics | حكومه | government |
| 28 | Politics | قانون | law |
| 29 | Politics | وزاره | ministry |
| 30 | Politics | برلمان | parliament |
| 31 | Politics | سياسه | politics |
| 32 | Politics | دوله | state |
| 33 | Religion | اسلام | Islam |
| 34 | Religion | صلاه | prayer |
| 35 | Religion | قران | Quran |
| 36 | Religion | مسجد | mosque |
| 37 | Religion | عباده | worship |
| 38 | Religion | حج | pilgrimage (Hajj) |
| 39 | Religion | زكاه | zakat (almsgiving) |
| 40 | Religion | رمضان | Ramadan |
| 41 | Sports | مباراه | match |
| 42 | Sports | كره | ball / football |
| 43 | Sports | بطوله | championship |
| 44 | Sports | لاعب | player |
| 45 | Sports | تدريب | training |
| 46 | Sports | فريق | team |
| 47 | Sports | ملعب | stadium |
| 48 | Sports | هدف | goal |
| 49 | Technology | اتصالات | telecommunications |
| 50 | Technology | حاسوب | computer |
| 51 | Technology | برنامج | program / software |
| 52 | Technology | انترنت | Internet |
| 53 | Technology | تقنيه | technology |
| 54 | Technology | هاتف | telephone |
| 55 | Technology | شبكه | network |
| 56 | Technology | بيانات | data |

**Table A.1.** The 56 benchmark queries by topical category.

Queries are listed in the normalized surface forms produced by the character-normalization pipeline described in Section 3.1 of the manuscript (taa marbuta mapped to haa, alef variants unified, alef maqsura mapped to yaa): for example, صلاه corresponds to the conventional orthography صلاة, and قران to قرآن. Each query is a single token; a document is judged relevant if it contains the query keyword or any inflectional surface variant of it (Section 3.6).
