# Supplementary material for: Arabic text preprocessing for dynamic searchable symmetric encryption: An empirical evaluation and preprocessor selection framework
Source: PLoS One. 2026 Sep 21;21(9):e0351281. doi: 10.1371/journal.pone.0351281 (PMC13592697; doi:10.1371/journal.pone.0351281)
Supplement: Supplementary file 2 [file pone.0351281.s002.docx]

**S2 Appendix. Pairwise Statistical Comparisons**

Supporting information for: "Arabic Text Preprocessing for Dynamic Searchable Symmetric Encryption: An Empirical Evaluation and Preprocessor Selection Framework" (PLOS ONE, Manuscript PONE-D-26-24849).

Table B.1 reports pairwise Wilcoxon signed-rank test results among the four stemmed preprocessors for end-to-end search latency (1,400 documents, 56 queries, 5 timed runs per query). Tests were conducted on per-keyword mean latencies (n = 56 paired observations per comparison) using SciPy 1.17.1 (scipy.stats.wilcoxon) under Python 3.12. Significance: * p < 0.05, ** p < 0.01, *** p < 0.001 (two-tailed, uncorrected).

All pairwise latency differences are statistically significant at p < 0.05, with most pairs at p < 0.001, confirming the performance ordering: NoPreprocessing (4.44 ms) < Farasa (5.35 ms) < Khoja (6.16 ms) < ISRI (10.31 ms) < Lucene (11.91 ms). Under a conservative Bonferroni correction for the ten pairwise comparisons reported in the study (α = 0.005), the Khoja–Farasa and ISRI–Lucene comparisons below are significant only at the uncorrected p < 0.05 level and should be interpreted with corresponding caution.

**Table B.1. Pairwise Wilcoxon signed-rank test results (search latency).**

| **Comparison** | **W-statistic** | **p-value** | **Significance** | **Faster** |
| --- | --- | --- | --- | --- |
| Khoja vs. ISRI | 153.5 | < 0.001 | *** | Khoja |
| Khoja vs. Lucene | 56.5 | < 0.001 | *** | Khoja |
| Khoja vs. Farasa | 526.0 | 0.041 | * | Farasa |
| ISRI vs. Lucene | 529.5 | 0.028 | * | ISRI |
| ISRI vs. Farasa | 47.5 | < 0.001 | *** | Farasa |
| Lucene vs. Farasa | 1.0 | < 0.001 | *** | Farasa |

**Search quality comparisons**

Table B.2 reports pairwise Wilcoxon signed-rank test results on per-query F1-scores across all five configurations, including the normalization-only baseline (n = 56 paired observations per comparison; two-tailed). Ten comparisons are reported; the Bonferroni-corrected threshold for this family is p < 0.005.

| **Comparison** | **W-statistic** | **p-value** | **Significance** | **Higher F1** |
| --- | --- | --- | --- | --- |
| NoPreprocessing vs. Khoja | 218.5 | < 0.001 | *** | NoPreprocessing |
| NoPreprocessing vs. ISRI | 204.0 | < 0.001 | *** | NoPreprocessing |
| NoPreprocessing vs. Lucene | 138.0 | 0.031 | ns† | Lucene |
| NoPreprocessing vs. Farasa | 174.0 | 0.092 | ns | Farasa |
| Khoja vs. ISRI | 562.0 | 0.790 | ns | ISRI |
| Khoja vs. Lucene | 18.0 | < 0.001 | *** | Lucene |
| Khoja vs. Farasa | 54.0 | < 0.001 | *** | Farasa |
| ISRI vs. Lucene | 53.0 | < 0.001 | *** | Lucene |
| ISRI vs. Farasa | 64.0 | < 0.001 | *** | Farasa |
| Lucene vs. Farasa | 122.0 | 0.424 | ns | Lucene |

Six of the ten quality differences are significant after Bonferroni correction. Both Lucene and Farasa significantly outperform both root-based stemmers. The Lucene–Farasa difference (p = 0.424) and the Khoja–ISRI difference (p = 0.790) are not significant, indicating that within each pair the two configurations are statistically indistinguishable in retrieval quality on this corpus.

**Table B.2.** Pairwise Wilcoxon signed-rank test results (search quality, per-query F1). Significance: *** p < 0.001; ns = not significant at the Bonferroni-corrected threshold (p < 0.005). † p = 0.031 is significant at the uncorrected 0.05 level but does not survive Bonferroni correction. “Higher F1” indicates which configuration has the larger mean F1, irrespective of significance.
